# Insight into gut dysbiosis of patients with inflammatory bowel disease and ischemic colitis

**DOI:** 10.3389/fmicb.2023.1174832

**Published:** 2023-05-11

**Authors:** Ram Hari Dahal, Shukho Kim, Yu Kyung Kim, Eun Soo Kim, Jungmin Kim

**Affiliations:** ^1^Department of Microbiology, School of Medicine, Kyungpook National University, Daegu, Republic of Korea; ^2^Department of Clinical Pathology, School of Medicine, Kyungpook National University, Daegu, Republic of Korea; ^3^Department of Internal Medicine, School of Medicine, Kyungpook National University, Daegu, Republic of Korea

**Keywords:** gut microbiome, inflammatory bowel disease, ischemic colitis, gut dysbiosis, biomarker, culturomics, uncultured gut bacteria, probiotics

## Abstract

The collection of whole microbial communities (bacteria, archaea, fungi, and viruses) together constitutes the gut microbiome. Diet, age, stress, host genetics, and diseases cause increases or decreases in the relative abundance and diversity of bacterial species (dysbiosis). We aimed to investigate the gut microbial composition at different taxonomic levels of healthy controls (HCs) with active Crohn’s disease (CD), ulcerative colitis (UC), and ischemic colitis (IC) using culture- and non-culture-based approaches and identify biomarkers to discriminate CD, UC, or IC. We determined the specific changes in the gut microbial profile using culture-independent (16S rRNA gene amplicon sequencing) as well as culture-based (culturomic) approaches. Biomarkers were validated using quantitative Real-Time PCR (qPCR). In both methods, bacterial diversity and species richness decreased in disease-associated conditions compared with that in HCs. Highly reduced abundance of *Faecalibacterium prausnitzii* and *Prevotella* sp. and an increased abundance of potentially pathogenic bacteria such as *Enterococcus faecium*, *Enterococcus faecalis*, and *Escherichia coli* in all CD, UC, or IC conditions were observed. We noted a high abundance of *Latilactobacillus sakei* in CD patients; *Ligilactobacillus ruminis* in UC patients; and *Enterococcus faecium*, *Escherichia coli*, and *Enterococcus faecalis* in IC patients. Highly reduced abundance of *Faecalibacterium prausnitzii* in all cases, and increased abundance of *Latilactobacillus sakei* and *Enterococcus faecium* in CD, *Ligilactobacillus ruminis* and *Enterococcus faecium* in UC, and *Enterococcus faecium*, *Escherichia coli*, and *Enterococcus faecalis* in IC could be biomarkers for CD, UC, and IC, respectively. These biomarkers may help in IBD (CD or UC) and IC diagnosis.

## Highlights

– Gut dysbiosis is influenced by the intestinal microbiota and inflammatory bowel disease (Crohn’s disease or ulcerative colitis) patients is characterized by depleted diversity of gut bacteria and short chain fatty acid (SCFA) producers such as *Faecalibacterium prausnitzii* and elevated abundance of proinflammatory bacteria such as adherent/invasive *Escherichia coli*.
– A comprehensive description of fecal microbial communities at different taxonomic levels in inflammatory bowel disease (both CD and UC) and ischemic colitis patients from both cultured-based and non-culture-based approaches.
– Highly reduced abundance of *Faecalibacterium prausnitzii* and increased abundance of *Enterococcus faecium*, *Escherichia coli*, and *Enterococcus faecalis* is characteristic sign of ischemic colitis.

## Introduction

The gut microbiota comprises bacteria, archaea, fungi, protozoa, and viruses. Gut microbiota disruption is called “gut dysbiosis” and is mainly influenced by intestinal microbiota, host genetics, host immunity, environmental factors, stress, diet, inflammation, and lifestyle (Wang et al., 2020). The gut microbiota can also be altered by the overuse or misuse of antibiotics (Belkaid and Hand, 2014). Gut dysbiosis is closely related to the pathogenesis of inflammatory disorders such as inflammatory bowel disease (IBD), colorectal cancer (CRC), multiple sclerosis (MS), rheumatoid arthritis (RA), and ischemic colitis (IC) (Yamashiro et al., 2017; Forbes et al., 2018; Wong and Yu, 2019). IBD has two major clinical forms, Crohn’s disease (CD) and ulcerative colitis (UC). It is a chronic inflammatory disorder affecting the gastrointestinal (GI) tract and is associated with significant morbidity, mortality, and costly medical care (Ananthakrishnan and McGinley, 2013). IBD has become a global public health challenge as the morbidity and mortality of IBD has constantly increased in recent years. Its incidence is increasing globally, and the highest rate has been noted in developed countries such as North America, Europe, Australia, and New Zealand, whereas it is much less common in Asia. However, in recent years, IBD has progressively become common in developed Asian countries, such as China, Japan, and South Korea (Yang, 2017). In Korea, the number of patients with UC and CD increased by 1.6- and 1.9-fold during the 2009–2016 period, respectively (Kwak et al., 2019).

Several efforts have been made to identify the pathogenesis of IBD. However, the precise etiology remains unclear, and a cure for IBD has yet to be discovered. Numerous studies have hypothesized the linkage of gut microbiota to inflammatory diseases (Nishida et al., 2018; Wang et al., 2020). Forbes et al. demonstrated that CD, UC, MS, and RA were associated with significantly altered gut microbiota composition (Forbes et al., 2018). Various studies have suggested that the structure and composition of the gut microbiota in IBD are extensively changed compared with those in HCs. Most alterations have been reported in the phyla *Firmicutes* and *Bacteroides. Faecalibacterium prausnitzii*, a member of the phylum *Firmicutes,* reportedly secretes anti-inflammatory metabolites and *Clostridium* spp. produce short-chain fatty acids (SCFA), and decreased abundance of these microbes has been reported in IBD (Caruso et al., 2020). In contrast, an increased abundance of *Enterococcus faecium*, *Escherichia coli*, and *Bacteroides fragilis* were noted in patients (Nagao-Kitamoto and Kamada, 2017; Seishima et al., 2019).

Ischemic colitis (IC) is another inflammatory disorder in the large intestine (inflammation in the colon owing to reduced blood flow) and is also associated with high morbidity and mortality (FitzGerald and Hernandez, 2015). Generally, IC incidence occurs in old age (>60 years), whereas IBD incidence is irrespective of the patient’s age. Peak incidence has been reported in the age range of 20–39 years, whereas over 15% of IBD cases have been diagnosed after the age of 60 years (FitzGerald and Hernandez, 2015; Nimmons and Limdi, 2016). Several attempts have been made to study gut dysbiosis between patients with IBD (UC and CD) and healthy individuals (Manichanh et al., 2012). However, human gut dysbiosis in IC compared with HCs has not yet been evaluated. Therefore, we attempted to identify the alterations in gut microbiome populations in IC patients compared with HCs in this study.

The human gut harbors 100 trillion diverse microbes, mostly (>99%) bacteria, over 1,100 prevalent species, and at least 160 different species that create a distinctive fingerprint in each individual (Qin et al., 2010). These different species in the gut play crucial roles in the immune system, regulation of intestinal function, nutrient digestion, metabolic homeostasis, and protection from pathogens. Additionally, specific gut bacterial species have been correlated with other bacterial infections (Buffie et al., 2015). Next-generation sequencing (NGS), metagenomics, metaproteomics, and metatranscriptomics can provide compositional information and some insights into the gut microbiota; however, determining the metabolism, characteristic features, phenotypes, enzymatic pathways, and ecological role of particular bacterial species without cultivation remains challenging. Additionally, various important biological roles remain to be discovered (Browne et al., 2016). Culturing unidentified species from the human gut microbiota will provide insight into the unprecedented biological role of new microbes as well as extend the gut taxonomic resolution. Moreover, culturing the gut microbiota from disease-associated individuals, such as IBD and IC, provides insights into gut microbial populations, species abundance, and characterization (phylotypes and pathotypes) of unknown species in addition to pathogenic or beneficial species of the gut.

We aimed to investigate the gut microbial composition of patients with active CD, UC, and IC compared with that in HCs using culture- and non-culture-based approaches, and identify the bacterial biomarker to discriminate CD, UC, and IC. Additionally, we aimed to cultivate previously uncultivated gut microbes from both disease-associated (IBD or IC) patients and healthy individuals. We successfully cultivated at least 100 novel species known to be previously uncultured bacterial clones. Notably, we explored the pathogenic species from IBD and IC fecal samples, which we hypothesized to be associated with disease promotion. Additionally, we identified potential novel probiotic bacterial strains that could be used in gut dysbiosis remission.

## Materials and methods

### Inflammatory bowel disease patients and HC group

We obtained 21 fecal samples from the Department of Laboratory Medicine, Kyungpook National University Hospital (KNUH), Daegu, South Korea. The fecal samples used in this study were residual samples used in diagnostic tests of patients in laboratory medicine. Among the 21 samples, 10 were from healthy people, and four, three, and four samples were from patients diagnosed with UC, CD, and IC, respectively. This study was approved by the Institutional Review Board (IRB) of Kyungpook National University Hospital (KNUH 2021–03–011-002).

### Isolation and identification of gut microbes from IBD patients and HC

Fresh fecal samples obtained from KNUH were immediately packed in an anaerobic gas pouch (BD GasPak EZ™ Pouch Systems, BD, NJ, United States) and transferred to an anaerobic gas chamber in the laboratory. One gram of each fecal sample was enriched in 9 ml of defibrinated sheep blood and incubated at 37°C for 2 days under aerobic and anaerobic conditions. The remaining fecal samples were stored at −70°C for further analysis. Serial dilutions of up to 10^9^ were made in phosphate-buffered saline (PBS), and 100 μl of each enriched fecal sample (10^5^–10^9^) was plated on 34 different defined agar plates (Supplementary Table S1) and incubated at 37°C for 3–10 days. Each colony on the agar plate was selected and streaked on a new agar plate until a pure colony was obtained. Purified colonies were kept at −70°C in 50% glycerol stock for long-term preservation. All isolated strains were identified using 16S rRNA gene sequencing by comparing the top-hit sequences of type strains from the EzBioCloud server^1^ or nucleotide BLAST from the NCBI database.^2^

### The 16S-based microbiome taxonomic profiling

Bacterial DNA was extracted from the fecal samples collected from each IBD patient and healthy individual using a FastDNA® spin kit for soil (MP Biomedicals, CA, United States) according to the manufacturer’s instructions. The sequence region of V3-V4 of the 16S rRNA bacterial gene was amplified using barcoded universal primers with the CFX 96 Real-Time system (c1000) (Bio-Rad, CA, United States) and KAPA SYBR FAST Universal 2× qPCR Master KAPA Biosystems (KAPA Biosystems, MA, United States). Amplicon Purification was conducted using cleanPCR (cleanNA, Netherlands), and the purified amplicon was quantified using the Quant-iT PicoGreen dsDNA Assay kit (Invitrogen, MA, United States). Bacterial libraries were prepared according to the 16S rRNA metagenomic sequencing library preparation protocol. The library quality was checked using an Agilent 2,100 Bioanalyzer System (Agilent Technologies). Sequencing was performed on an Illumina MiSeq platform using the MiSeq Reagent Kit v2 (500-cycles) at ChunLab Inc. (Republic of Korea). The sequence data were processed and analyzed following the NGS Analyses Manual (QIIME) (Caporaso et al., 2010) and the 16S-based microbiome taxonomic profiling platform of EzBioCloud Apps and analyzing system.^3^ All the chimeras were removed from the raw data (FATSQ) and further filtered and trimmed using CD-HIT-OTU and rDNA tools (Schloss et al., 2009; Li et al., 2012). Finally, assembled reads were clustered into operational taxonomic units (OTUs) using the greedy algorithm at a cut-off value of 97% identity at the species level (Rideout et al., 2014). All the representative sequences from each OTU were used for taxonomic assignment, and phylum-to-species level characterization was performed using the RDP-16S rRNA reference database^4^ and the UCLUCT program (Edgar and Bateman, 2010). An expression-based heatmap was generated based on the complete linkage of clustering and Pearson’s distance measurement methods using a heat mapper (Babicki et al., 2016). Unprocessed pair-end sequence reads were deposited in the Sequence Read Archive (SRA) under BioProject ID: PRJNA884507.

### Quantitative real-time PCR amplification

For quantitative real-time PCR (qPCR), fecal bacterial DNA was extracted using a FastDNA® spin kit for soil (MP Biomedicals, CA, United States), according to the manufacturer’s instructions. Extracted DNA was stored at −20°C until analysis. Quantification of the 16S rRNA genes of *Faecalibacterium prausnitzii*, *Latilactobacillus sakei*, *Ligilactobacillus ruminis*, *Escherichia coli*, *Enterococcus faecalis*, *Enterococcus faecium*, and total bacteria was determined by quantitative real-time polymerase chain reaction (qPCR). Amplification was performed using an ABI StepOnePlus Real-Time PCR System (Applied Biosystems, MA, United States). The reaction mixture (20 μl) was prepared using TOPreal™ q-PCR 2× PreMIX (SYBR Green with high ROX, Enzynomics), 10 μl; forward primer (10 pmol/μL), 1 μl; reverse primer (10 pmol/μL), 1 μl; and 12 ng of template DNA, 1 μl. The amplification program consisted of pre-denaturation at 95°C for 10 min, followed by 40 cycles of 95°C for 15 s, 60°C for 1 min, 72°C for 30 s, and a final cycle of 95°C for 15 s. All primers used in this study for each gene are listed in Supplementary Table S2. Melting curves were obtained and inspected after amplification to determine the specificity of PCR. The expression levels were normalized to the levels of the housekeeping 16S rRNA gene. The fold change in gene expression was determined using the ΔΔC_T_ method. The experiments were performed in triplicate.

### Statistical analyses

The Wilcoxon rank-sum test was used to compare the differential abundance analysis of annotated taxa. Alpha diversity (species richness: ACE, Chao 1, Jackknife, and number of OTU; diversity index: NPShannon, Shannon, Simpson, and phylogenetic diversity; and other statistics related to species abundance and evenness, including rarefaction curve) and beta diversity (principal coordinate analysis and UPGMA clustering) were analyzed using OTU information from the EzBioCloud MTP pipeline^5^. The mean, standard error (SE), standard deviation (SD), one-way analysis of variance (ANOVA), and Tukey’s honest significance tests were performed using OriginPro8.5 (OriginLab, MA, United States). In all analyses, *p* < 0.05 was regarded as statistically significant.

## Results

### Microbial composition in IBD patients and HCs based on 16S-based MTP at different taxonomic levels

**Figure 1:**
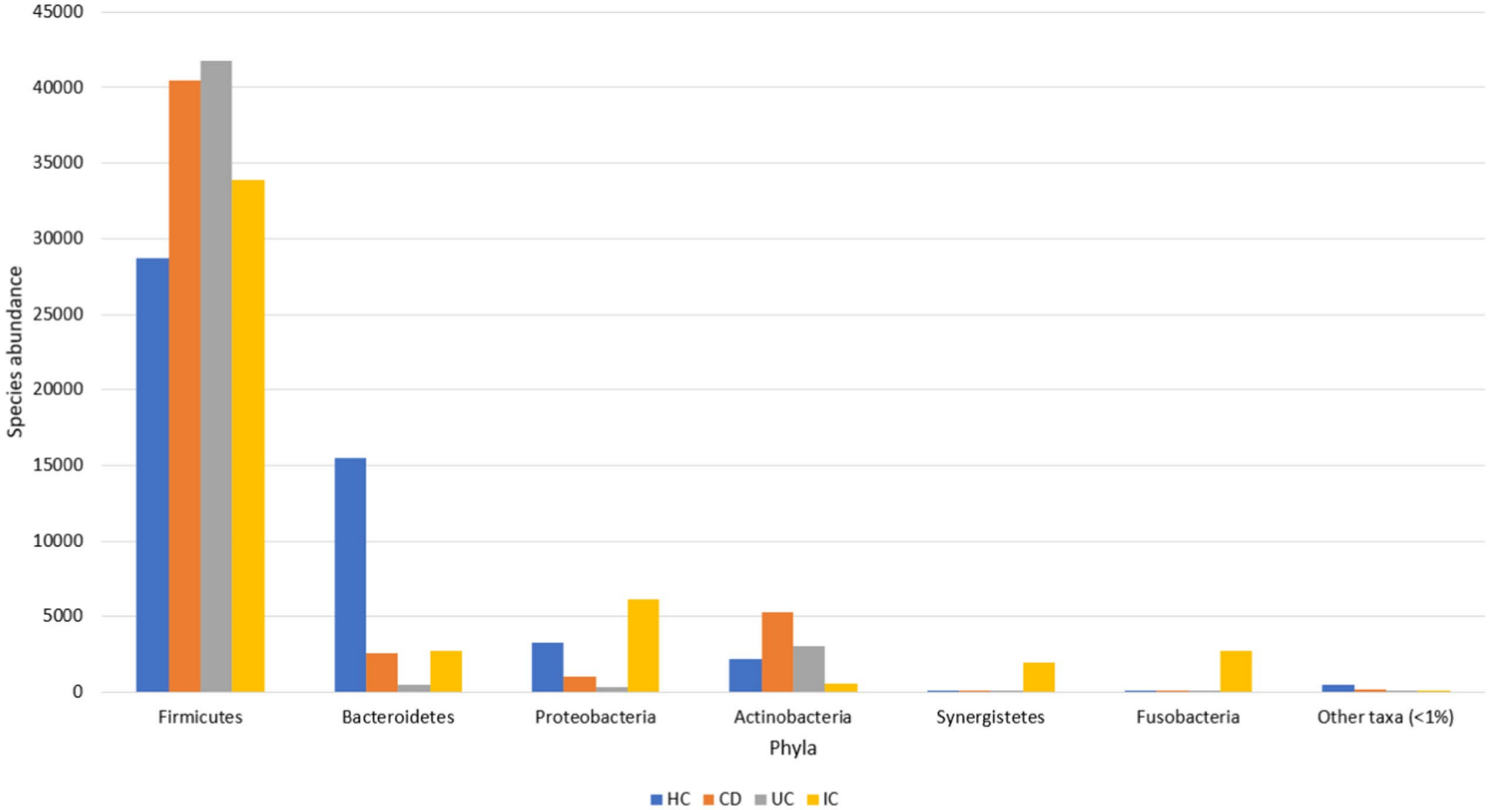
Gut microbial abundance in different conditions at the phylum level. Phylum level abundance in HC, CD, UC, and IC.

The major gut microbial abundances observed in IBD samples were *Firmicutes* (87.1%), *Actinobacteria* (8.5%), *Bacteroidetes* (2.9%), and *Proteobacteria* (1.3%), followed by other taxa (<1%) (Supplementary Figure S1). In contrast, the principal abundances in the HCs were *Firmicutes* (57.2%), *Bacteroidetes* (30.9%), *Proteobacteria* (6.5%), and *Actinobacteria* (4.4%), followed by other taxa (<1%) (Figure 1). Other phyla observed in the IBD samples were *Acidobacteria*, candidate bacterial phyla (BRC1), *Chlamydiae*, *Chlorobi*, *Chloroflexi*, *Cyanobacteria*, *Deinococcota*, *Gemmatimonadetes*, *Kazan*, *Lentisphaerae*, *Nitrospirae*, *Parcubacteria* (OD1), *Planctomycetes*, *Rhodothermaeota*, *Saccharibacteria* (TM7), *Synergistetes*, candidate bacterial phyla (TM6), *Tenericutes*, and *Verrumicrobia*. Similarly, other genera identified in the HCs were *Acidobacteria*, *Chloroflexi*, *Fusobacteria*, *Gemmatimonadetes*, *Lentisphaerae*, *Nitrospirae*, *Planctomycetes*, *Saccharibacteria* (TM7), *Synergistetes*, *Tenericutes*, and *Verrumicrobia*. Some phyla, such as BRC1, *Chlamydiae*, *Chlorobi*, *Cyanobacteria*, *Deinococcota* (*Deinococcus*-*Thermus*), *Kazan*, OD1, *Rhodothermaeota*, and TM6, observed in the IBD samples were not observed in HC samples (Supplementary Figure S1b).

The dominant phyla under both conditions (IBD and HC) were *Firmicutes*, *Actinobacteria*, *Bacteroidetes*, and *Proteobacteria*, whereas *Firmicutes* was the most abundant phylum in both IBD and HC samples. However, the abundance of *Firmicutes* was at least 29% higher in the IBD group than in the HCs (Supplementary Figure S1a). Compared with HC, in CD and UC, the abundance of *Firmicutes* increased 1.4- and 1.6-fold, respectively (Supplementary Figure S1a). Species abundance of the phylum *Firmicutes* in UC was 1.2-fold higher than that in CD (Supplementary Figure S1a). The relative abundance of *Firmicutes* in the IBD samples was statistically significant (*p* < 0.001) compared with that in the HCs (Supplementary Figure S2). Additionally, the abundance of *Actinobacteria* in the IBD condition was increased by over 1.9-fold. The abundance of *Actinobacteria* in CD and UC was increased by 2.4- and 1.5-fold, respectively, whereas the abundance in CD was 1.6-fold higher than that in UC (Supplementary Figure S1a). No significant differences were observed in the *Actinobacteria* levels between HC, CD, and UC. Notably, the abundance of *Proteobacteria* decreased 4.9-fold compared with that in controls. In contrast, the abundance of *Proteobacteria* in HC was almost 3- and 9.3-fold higher than that in CD and UC, respectively. However, the abundance in CD was increased by 3.1-fold compared with that in UC (Supplementary Figure S1a). Moreover, the abundance of *Bacteroidetes*, a phylum associated with healthy gut microbiota, was sharply decreased in the IBD condition by 10.6-fold (Supplementary Figure S1a). The abundance in CD and UC was decreased 6- and 30.9-fold, respectively, compared with those in the HC group. Conversely, the abundance in UC was further decreased by 5.1-fold compared with that in the CD group (Supplementary Figure S1a). The relative abundance of *Bacteroidetes* in the HC against IBD condition was statistically significant (*p* < 0.001) (Supplementary Figure S3).

**Figure 2:**
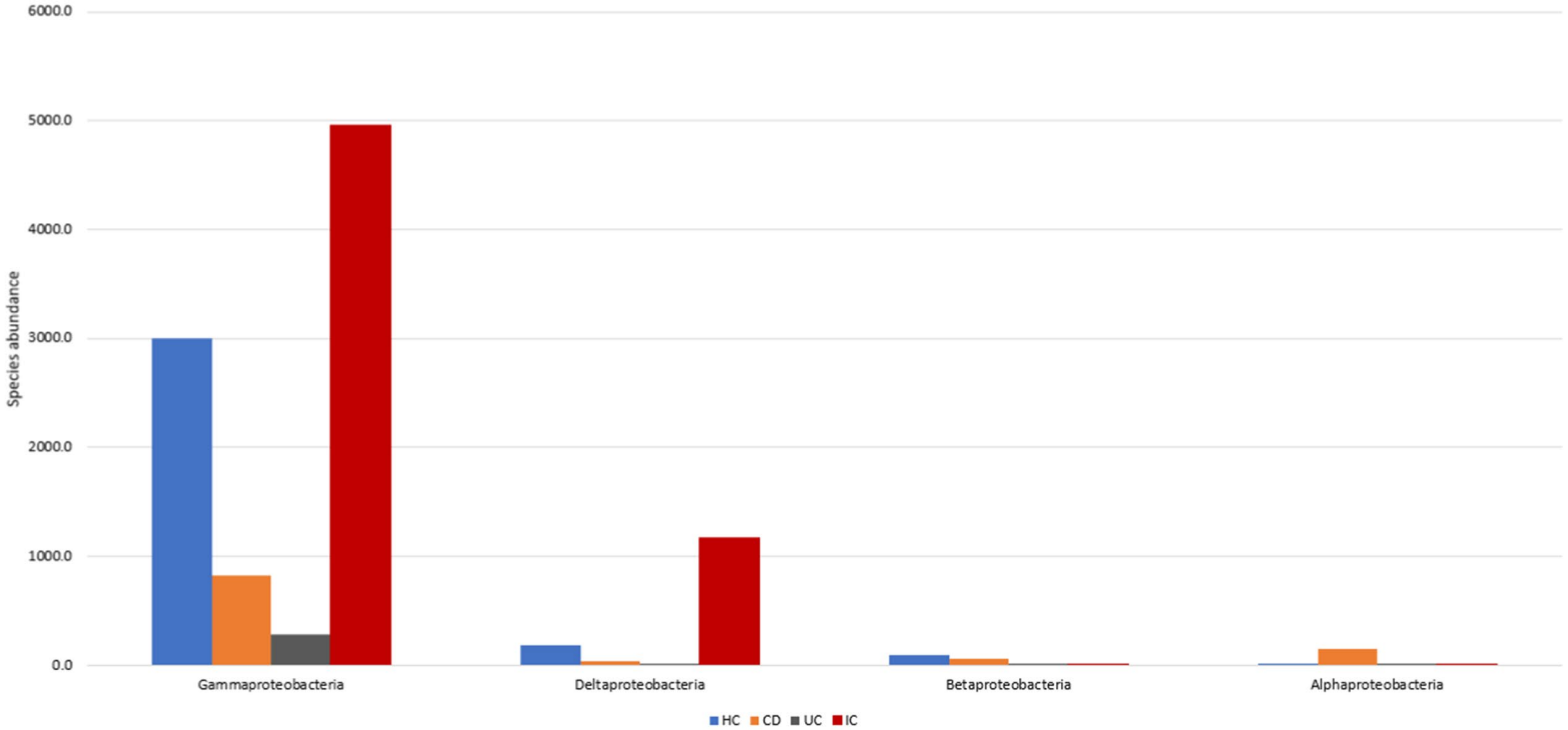
*Proteobacteria* abundance in HC, CD, UC, and IC.

Focusing on these four phyla, we further investigated microbial abundance at the class, family, and species levels. In patients with IBD, the abundance of four classes *of Erysipelotrichia*, *Actinobacteria*, *Bacilli,* and *Alphaproteobacteria* increased 1.5-, 2.6-, 9.4-, and 34.3-fold, respectively. In contrast, the abundance of five classes *of Coriobacteriia*, *Tissierellia*, *Clostridia*, *Bacteroidia,* and *Negativicutes* decreased 1.3-, 1.3-, 3-, 12.0-, and 24.6-fold, respectively, compared with those in HCs (Supplementary Figures S4a,b). High fluctuations in abundance in classes *Alphaproteobacteria* and *Negativicutes* were observed. Individually, the abundance of class *Alphaproteobacteria* in CD and UC patients was increased by 77.4 and 2-fold against HCs, and the abundance ratio for CD to UC was 735:19 (Figure 2). In contrast, the class abundance of *Negativicutes* in CD and UC patients was lowered 49.9- and 17.9-fold compared with those in HC (Supplementary Figures S4a,b). The abundance in CD was decreased 2.8-fold compared with that in the UC condition (Supplementary Figures S4a,b). In the phylum *Proteobacteria*, *Gammaproteobacteria* was observed to be the most abundant class compared to *Deltaproteobacteria*, *Betaproteobacteria*, and *Alphaproteobacteria* (Figure 2).

The abundance of 13 families, including *Ruminococcaceae*, *Lachnospiraceae*, *Bacteroidaceae*, *Enterobacteriaceae*, *Clostridiaceae*, *Coriobacteriaceae*, *Acidaminococcaceae*, *Pasteurellaceae*, *Veillonellaceae*, *Christensenellaceae*, *Peptoniphilaceae*, *Desulfovibrionaceae*, and *Mogibacteriaceae*, decreased in IBD conditions. Conversely, the abundance of 11 families, including *Streptococcaceae*, *Peptostreptococcaceae*, *Erysipelotrichaceae*, *Lactobacillaceae*, *Enterococcaceae*, *Eubacteriaceae*, *Actinomycetaceae*, *Aerococcaceae*, *Oxalobacteriaceae*, *Xanthomonadaceae*, and *Morganellaceae*, increased in IBD patients compared with that in HCs (Supplementary Figures S5a,b). The most abundant family in the HC was *Ruminococcaceae*. The abundance in the IBD, UC, and CD conditions was decreased 8.7-, 6.2-, and 21.4-fold, respectively, than in HCs, whereas the abundance in CD was decreased 3.5-fold than in the UC condition (Supplementary Figure S5b). Similarly, the second most abundant family in HC was *Lachnospiraceae*. The abundance in IBD, CD, and UC conditions decreased 3.8-, 4.0-, and 3.8-fold, respectively, and the abundances in both UC and CD conditions were similar (Supplementary Figures S5a,b). In contrast, the abundance of two families, *Lactobacillaceae* and *Enterococcaceae*, increased 67.9- and 22.4-fold, respectively, in IBD patients compared with that in the healthy individuals. Moreover, the abundance of *Lactobacillaceae* in CD and UC conditions increased 55.6- and 77.0-fold compared with that in HC, whereas the average abundance of UC to CD conditions was 302,335:3928 (Supplementary Figure S5b). Similarly, the abundance of *Enterococcaceae* in UC and CD fecal samples increased 11.0- and 37.5-fold, respectively, compared with that in the control group. The abundance in UC was more than 3.4-fold compared with that in the CD condition (Supplementary Figure S5a). Additionally, the abundance of the families *Streptococcaceae* and *Peptostreptococcaceae* under IBD conditions also increased by 1.1- and 2.2-fold, respectively (Supplementary Figures S5a,b).

At the genus level, *Bacteroides*, *Faecalibacterium*, and *Prevotella* were the most abundant in HC. Their abundance decreased 7.9-, 39.9-, and 231.6-fold, respectively, under IBD conditions (Supplementary Figures S6a,b). Surprisingly, in the UC condition, the abundance of *Faecalibacterium*, *Prevotella*, and *Bacteroides* was reduced by 699.7-, 172.8-, and 20.9-fold, respectively, compared with that in HCs (Supplementary Figures S6a,b). For the genus *Prevotella* alone, the abundance decreased 420.9-fold in CD compared with that in HC. The relative abundance based on Wilcoxon rank-sum with the genera *Faecalibacterium* and *Bacteroides* were statistically significant for both UC and CD conditions than in HCs (Supplementary Figure S7). Additionally, decreased abundance of *Clostridium*, *Blautia*, *Megamonas*, *Eubacterium*_g23, *Eubacterium*_g5, *Roseburia*, *Oscillibacter*, *Lachnospira*, *Catenibacterium*, *Phascolarctobacterium*, *Holdemanella*, *Subdoligranulum*, *Haemophilus*, *Alistipes*, *Clostridium*_g24, *Dorea*, *Collinsella*, *Ruminococcus*_g4, *Ruminococcus*, *Agathobacter*, *Dialister*, *Fusicatenibacter*, *Paraprevotella*, *Anaerostipes*, *Sporobacter*, *Agathobaculum*, *Ruminococcus*_g2, *Senegalimassilia*, *Pseudoflavonifractor*, *Weissella*, *Bilophila*, *Caproiciproducens*, *Desulfovibrio, Sutterella*, *Citrobacter*, *Barnesiella*, *Paludicola*, and *Mogibacterium* were observed in IBD’s fecal samples than that of HCs. In contrast, we observed an increased abundance of the genera *Lactobacillus*, *Streptococcus*, *Peptostreptococcus*, *Bifidobacterium*, *Romboutsia*, *Enterococcus*, *Longicatena*, *Intestinibacter*, *Eggerthella*, *Turicibacter*, *Sellimonas*, *Clostridium*_g6, *Merdibacter*, *Escherichia*, *Pediococcus*, *Eubacterium*, *Actinomyces*, *Clostridioides*, *Fournierella*, *Merdibacter*, and *Faecalimonas* in IBD patients than in HCs (Supplementary Figures S6a,b). Particularly, the abundance of 12 genera, including *Lactobacillus*, *Streptococcus*, *Bifidobacterium*, *Romboutsia*, *Enterococcus*, *Longicatena*, *Intestinibacter*, *Eggerthella*, *Turicibacter*, *Sellimonas*, *Merdibacter*, and *Escherichia*, increased 64.4-, 1.1-, 2.4-, 1.8-, 22.4-, 31.4-, 9.3-, 33.3-, 6.7-, 11.3-, 2,294-, and 1.01-fold, respectively (Supplementary Figure S8). The relative abundances of *Lactobacillus*, *Bifidobacterium*, *Romboutsia*, *Enterococcus* were statistically significant in IBD condition when compared to HC (Supplementary Figure S9). In addition, statistically significant species abundance was observed in the genera *Intestinibacter*, *Eggerthella*, *Sellimonas*, or *Merdibacter* during IBD condition than HC. However, no statistically significant association was observed in the genera *Streptococcus*, *Longicatena*, or *Turicibacter* between IBD and HC.

At the species level, the most abundant species in healthy individuals was *Faecalibacterium prausnitzii*, and the abundance in CD and UC patients was decreased 22.7- and 91.6-fold, respectively, followed by uncultured *Prevotella* (PAC001304_s), which was decreased by 430.3- and 384.4-fold in CD and UC patients, respectively (Supplementary Figures S10a,b). Moreover, the abundance of species such as *Clostridium celatum*, uncultured *Prevotella* (*Prevotella*_uc), *Bacteroides coprocola*, *Megamonas rupellensis*, *Bacteroides vulgatus*, *Bacteroides uniformis*, *Blautia wexlerae*, uncultured *Eubacterium* species (PAC001051_s), *Bacteroides dorei*, *Eubacterium hallii*, *Bifidobacterium adolescentis*, *Holdemanella biformis*, *Roseburia inulinivorans*, *Bacteroides ovatus*, *Prevotella copri*, *Ruminicoccus callidus*, and *Dorea longicatena* was significantly reduced in the IBD group (Supplementary Figure S10a). The abundance of beneficial *Akkermansia muciniphila*, a member of the phylum *Verrumicrobia*, was much lower in patients with UC and CD (51.1- and 1,120-fold, respectively) than in HC. In contrast, the abundance of two species, *Ligilactobacillus ruminis* and *Latilactobacillus sakei*, was substantially increased by 1,039 and 33.9-fold, respectively, under IBD conditions (Supplementary Figure S11). Additionally, the abundance of *Ligilactobacillus ruminis* in CD and UC was 1.9- and 1816.8-fold higher than that in HC. The abundance of *Latilactobacillus sakei* in CD patients increased significantly (79.1-fold). However, it was decreased in UC patients (32-fold) compared with that in HCs (Supplementary Figure S11). Furthermore, the abundance of *Romboutsia timonensis*, *Lactobacillus paracasei* group, *Clostridium innocuum* group, *Intestinibacter bartlettii*, *Turicibacter sanguinis*, *Streptococcus sinensis*, *Pediococcus acidilactici*, *Escherichia coli*, *Streptococcus pneumoniae*, *Clostridioides difficile*, *Fournierella massiliensis*, *Enterococcus faecalis*, and *Morganella morganii* groups was increased in IBD patients compared with that in HCs (Supplementary Figures S10, S11). We compared the *relative abundance of Enterococcus faecium* at the species level in the IBD and control groups. A statistically significant difference was observed between patients with IBD and HCs (*p* < 0.05) (Supplementary Figure S12). These results suggested that a high abundance of *Enterococcus faecium* might promote IBD pathogenesis. Seishima et al. (2019) reported that human gut-derived *Enterococcus faecium* is vital in promoting colitis in genetically susceptible mice. However, comprehensive studies on disease progression caused by these bacteria should be conducted.

### Microbial composition in IC and healthy control groups based on 16S-based MTP at different taxonomic levels

For the taxonomic composition at the phylum level, *Firmicutes* were the most abundant in both groups (IC and HC), and the abundance in the IC condition was 1.2-fold higher than that in HC. The individual phyla present in the fecal samples of patients with IC were *Firmicutes*, *Proteobacteria*, *Bacteroidetes*, *Fusobacteria*, *Synergistetes*, *Actinobacteria*, *Tenericutes*, *Saccharibacteria*_TM7, *Acidobacteria*, *Chloroflexi*, *Verrumicrobia*, *Gemmatimonadetes*, and *Nitrospirae,* where the species abundance of the first six phyla accounted for more than 99.9% (Supplementary Figure S1). The abundance of *Proteobacteria*, *Synergistetes*, and *Fusobacteria* increased 1.9-, 42.9-, and 775.3-fold in IC patients, whereas the decreased abundance of *Verrumicrobia*, *Bacteroidetes*, and *Tenericutes* were 3.8-, 5.6-, and 20.9-fold, respectively (Supplementary Figures S1, S13). For IC patients, the abundance of eight classes, including *Bacilli*, *Clostridia*, *Gammaproteobacteria*, *Bacteroidia*, *Fusobacteria*, *Synergistia*, *Deltaproteobacteria*, and *Erysipelotrichia* accounted for >98% (Supplementary Figures S4a, S13b). Conversely, the abundance of five classes, *Gammaproteobacteria*, *Bacilli*, *Deltaproteobacteria*, *Synergistia*, and *Fusobacteria*, increased by 1.7-, 5.6-, 6.6-, 42.9-, and 775.1-fold, respectively. A significant association exists between IC and HC microbial abundance of the *Bacilli*, *Gammaproteobacteria*, *Fusobacteria*, and *Bacteroidia* classes (Supplementary Figure S14).

The microbial composition at the family level during IC revealed a high abundance in the family *Enterococcaceae* (37%), followed by *Enterobacteriaceae* (10.3%), *Peptostreptococcaceae* (7.7%), *Lachnospiraceae* (6.7%), *Fusobacterium* (5.6%), *Clostridiaceae* (4.6%), *Ruminococcaceae* (4.5%), *Synergistaceae* (4.1%), *Streptococcaceae* (3.4%), *Desulfovibrionaceae* (2.4%), *Rikenellaceae* (2.1), and *Eubacteriaceae* (1.9%), which accounted for over 92% of the total gut microbiota detected thus far (Supplementary Figure S13c). During IC, the first three most abundant families, *Enterococcaceae*, *Enterobacteriaceae*, and *Peptostreptococcaceae*, increased 193.6-, 1.9-, and 2.9-fold, respectively, whereas the three most abundant families, *Ruminococcaceae*, *Lachnospiraceae*, and *Bacteroidaceae*, in HC were decreased 4.4-, 2.4-, and 8.8-fold, respectively, in IC fecal samples. A significant association was observed for relative abundance between these families in IC and HCs (Supplementary Figures S5a, S15). When we observed the species abundance at the genus level in the fecal samples of IC, the most abundant genera were *Enterococcus* (37.1%), followed by *Escherichia* (7.3%), *Fusobacterium* (5.6%), *Clostridium* (4.6), *Pyramidobacter* (4.1%), *Romboutsia* (3.6%), *Streptococcus* (3.4%), *Clostridium*_g35 (3.3%), *Enterobacteriaceae_*g (2.3%), *Alistipes* (2.1%), *Ruminococcus*_g2 (1.7%), and *Lactococcus* (1.7%), accounting for >76% (Supplementary Figure S13). Additionally, highly increased abundance of the genus such as *Clostridioides* (2221.5-fold), *Fusobacterium* (1595.6-fold), *Pyramidobacter* (846.9-fold), *Paraclostridium* (378.9-fold), *Enterococcus* (193.6-fold), *Eubacterium* (51.2-fold), *Escherichia* (16.4-fold), *Intestinibacter* (12.7-fold), *Bilophila* (7.5-fold), *Longicatena* (7.4-fold), *Desulfovibrio* (7.2-fold), *Eggerthella* (4.6-fold), *Alistipes* (2.4-fold), and *Romboutsia* (1.5-fold) was observed in IC fecal samples than that of HCs. Conversely, highly decreased abundance was observed in *Megamonas* (3236.2-fold), *Catenibacterium* (1003.4-fold), *Akkermansia* (674.4-fold), *Prevotella* (537.8-fold), *Holdemanella* (441.6-fold), *Weissella* (424-fold), *Dorea* (39.7-fold), *Eubacterium*_g23 (32.3-fold), *Lachnospira* (27.6-fold), *Faecalibacterium* (26.1-fold), *Paraprevotella* (15.4-fold), *Agathobacter* (10.1-fold), *Bacteroides* (8.8-fold), *Collinsella* (6.8-fold), *Bifidobacterium* (5.8-fold), *Blautia* (3.7-fold), and *Parabacteroides* (1.4-fold) (Supplementary Figures S6a, S13d).

At the species level abundance, 14 species, including *Enterococcus faecium* (293.4-fold), *Escherichia coli* (16.4-fold), *Fusobacterium nucleatum* (1798-fold), *Pyramidobacter piscolens* (846.9-fold), *Romboutsia timonensis* (1.5-fold), *Clostridium symbiosum* (345-fold), *Intestinibacter bartlettii* (12.7-fold)*, Paraclostridium benzolyticum* group (378.9-fold), *Bilophila wadsworthia* (7.5-fold), *Alistipes onderdonkii* (7.5-fold), *Clostridioides difficile* group (2221.5-fold), *Enterococcus faecalis* (13.1-fold), *Lactobacillus salivarius* (331.4-fold), and *Lacticaseibacillus paracasei* group (31.8-fold) were most abundant comprising a total of 71% of gut microbiota. The respective abundance was increased during IC than that of healthy individuals (Supplementary Figures S16a–c). The abundances of *Finegoldia magna*, *Fusobacterium varium* group, and *Longicatena caecimuris* were increased by 40.4-, 50-, and 30-fold, respectively. In contrast, a decreased abundance of highly beneficial species, including *Faecalibacterium prausnitzii* (25.9-fold) and *Akkermansia muciniphila* (672.2-fold), was observed during IC (Supplementary Figure S17a). Additionally, reduced abundance also observed in 141 species including the following: uncultured *Prevotella* (PAC001304-s, *Prevotella*_uc, and PAC001292_s; 6029.1-fold), *Bacteroides coprocola* (3261-fold), *Megamonas rupellensis* (3134.8-fold), uncultured *Bacteroidales* (PAC001134_s; 1639.7-fold), uncultured *Catenibacterium* (PAC002523_s; 1,003-fold), uncultured *Eubacterium* (PAC001051_s, PAC001033_s, and PAC001035_s; 990.5-fold), *Anaerococcus vaginalis* (423.7-fold), *Prevotella copri* (357.2-fold), *Bacteroides plebeius* (325-fold), *Bacteroides intestinalis* (247.3-fold), *Anaerococcus prevotii* (233-fold), *Bacteroides dorei* (200.7-fold), *Weissella kandleri* (181.3-fold), *Eubacterium eligens* (147.5-fold), *Agathobaculum butyriciproducens* (96.7-fold), *Prevotella stercorea* (93.9-fold), *Blautia luti* (71.9-fold), *Ruminococcus lactaris* (60.8-fold), *Gemmiger formicilis* (56.3-fold), *Eubacterium hallii* (16.1-fold), *Bifidobacterium longum* (15.1-fold), *Paraprevotella clara* (13.2-fold), *Ruminococcus faecis* (12.3-fold), *Bacteroides uniformis* (7.4-fold), *Collinsella aerofaciens* (6.8-fold), *Blautia wexlerae* (4.2-fold) (Supplementary Figure S17).

### Alpha and beta diversity of CD, UC, IC, and HC groups

**Figure 3:**
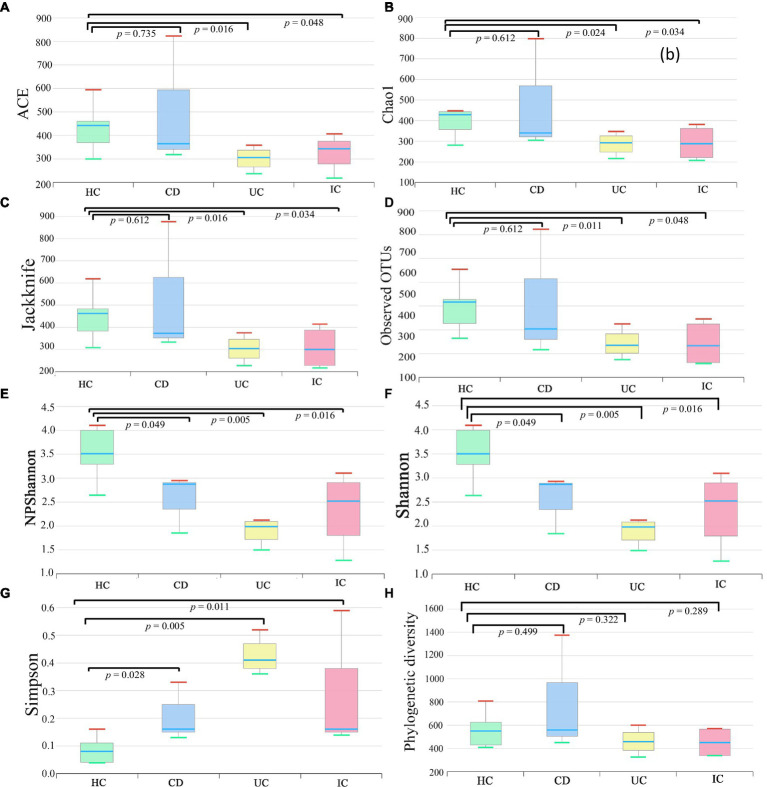
Alpha (species richness and evenness) diversity analysis of HC, CD, UC, and IC samples. Within-sample diversity was measured by indices: ACE **(A)**, Chao1 **(B)**, Jackknife **(C)**, observed OTUs **(D)**, NPShannon **(E)**, Shannon **(F)**, Simpson **(G)**, and Faith’s phylogenetic diversity **(H)**. Wilcoxon rank-sum test was performed to analyze statistical significance.

**Figure 4:**
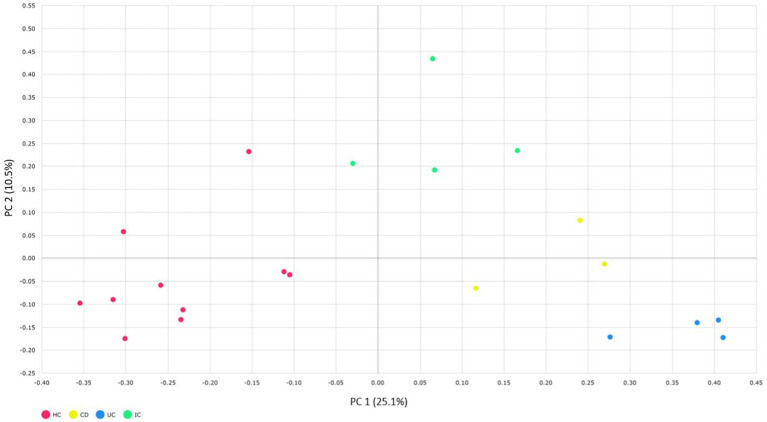
Beta diversity of CD, UC, IC, and HC principal coordinate analysis of the generalized UniFrac distances for healthy control and CD, UC, and IC conditions (principal component 1 versus 2). Percentages (%) = variance explained. Permutational multivariate analysis of variance (PERMANOVA) was performed to analyze statistical significance.

We compared the alpha and beta diversities in the IBD (CD and UC), IC, and HC groups. Differences in within-sample phylotypes, species richness, and evenness were observed between HCs (HC) and disease-associated samples (CD, UC, and IC) (Figure 3). The rarefaction curve plotted against OTU and the total reads of each taxonomic profile revealed that the highest number of reads was from HC samples than from disease-association (IBD or IC) samples (Supplementary Figure S18). Beta diversity distance was calculated based on generalized UniFrac divergence, and species rank, including unclassified OTUs and reads, was used. Principal coordinate analysis revealed four distinct samples (HC, CD, IC, and UC) clustered based on sample types, either healthy control or diseased condition (IBD and IC). Disease-associated samples were located furthest from HCs, and statistically significant dissimilarities between healthy control and disease association were observed in generalized UniFrac diversity distance (HC to CD, *p* = 0.004; HC to UC, *p* = 0.001; and HC to IC, *p* = 0.001) (Figure 4). The phylogenetic distances of the CD, UC, and IC groups and HCs revealed that the taxonomic profile of disease-associated groups was different from that of HCs, as demonstrated by UPGMA clustering. All HCs were clustered together, and the taxonomic profiles of the CD, UC, and IC groups clustered to form a different clade (Supplementary Figure S19). Finally, expression level heatmap (at species level) also supported the grouping of disease-associated samples against healthy controls which clearly differentiated the CD, UC, or IC with separate cluster, respectively (Supplementary Figure S20).

### Microorganisms isolated from disease-associated fecal samples (CD, UC, and IC)

A total of 1,032 (CD, *n* = 274; UC, *n* = 354; and IC, *n* = 404) pure cultures were isolated from 11 fecal samples of disease-associated human fecal samples using 34 different types of culture media, both under aerobic and anaerobic conditions, which comprised five phyla (*Firmicutes*, *Proteobacteria*, *Actinobacteria*, *Bacteroidetes*, and *Fusobacteria*), 30 families, 50 genera, and 106 different species (Supplementary Figure S21 and Supplementary Table S3). Based on the culturomic data, the dominant phylum was *Firmicutes* (61.4%), followed by *Proteobacteria*, *Actinobacteria*, *Bacteroidetes*, and *Fusobacteria*. Out of 1,032 isolated strains, 32 isolates shared <98.9% of 16S rRNA gene sequence similarities to the type species of various genera, indicating potential novel species, and eight bacteria were previously uncultured bacterial isolates. The 16S rRNA gene sequence similarity between 98.7 and 99.2% supports the proposal for novel species (Bosshard et al., 2003; Yarza et al., 2014). Potential novel species isolated from disease-associated fecal samples (CD, UC, or IC) are presented in Supplementary Table S4.

We explored novel species from the human gut as well as reported some species from the human gut microbiota for the first time. *Kalamiella piersonii* (*n* = 111; 11% of total isolates; Supplementary Table S3) was the second most dominant isolate from 11 disease-associated fecal samples, which were initially isolated from an international space station environmental sample by Singh et al. (2019). Notably, neither this species nor genus was observed in the 16S-based MTP profile. Furthermore, we report species such as *Bacillus tequilensis*, *Bacillus hisashii, Dietzia cercidiphylli*, *Enterococcus hulanensis*, *Hymenobacter glaciceicola*, *Lysinibacillus capsici*, *Nocardioides rotundus*, *Paraclostridium benzoelyticum Proteus terrae*, *Pseudonocardia carboxydivorans*, *Rhodococcus qingshengii*, *Staphylococcus petrasii* subsp. *pragensis*, *Streptococcus australis*, and *Weissella koreensis*, which were isolated from disease-associated fecal samples (CD, UC, or IC) for the first time from the human gut.

### Microorganisms isolated from HCs

Similarly, from the 10 fecal samples from healthy individuals, a total of 1,206 pure cultures were isolated, which consisted of six phyla (*Actinobacteria*, *Bacteroidetes*, *Firmicutes*, *Fusobacteria*, *Proteobacteria*, and *Verrumicrobia*), 12 classes (*Actinomycetia*, *Coriobacteriia*, *Bacteroidia*, *Clostridia*, *Bacilli*, *Erysipelotrichia*, *Negativicutes*, *Tissierellia*, *Fusobacteriia*, *Gammaproteobacteria*, *Betaproteobacteria*, *and Verrumicrobiae*), 41 families, 87 genera, and 174 different species, excluding potential novel species (Supplementary Figure S21 and Supplementary Table S5). From fecal samples of HCs, we successfully cultured 213 isolates of previously uncultured species from the human gut microbiota. The 16S rRNA gene sequence similarity of the 15 isolates was <95%, indicating potential novel genera (Supplementary Table S6; Bosshard et al., 2003).

**Figure 5:**
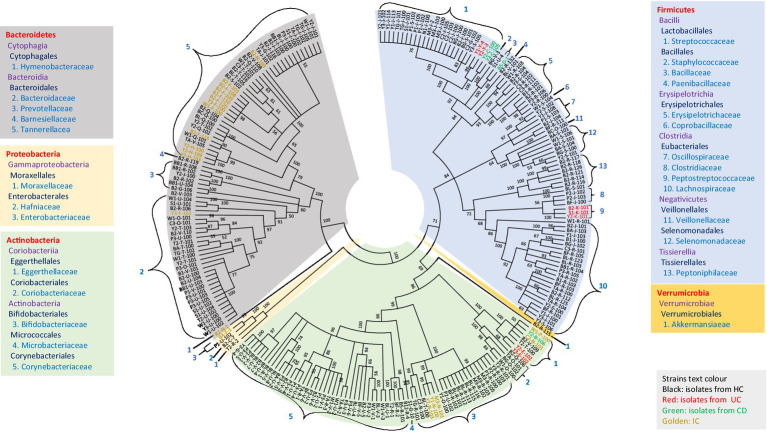
Diversity of novel gut isolates from disease-associated (CD, UC, and IC) and HC fecal samples. The cladogram represents the taxonomic classification of all 245 potential novel strains comprising at least 100 novel species from 27 families down to the family level. The cladogram is color-coded, as depicted in the boxes according to five phyla. The text color of the strains demonstrates the sources of isolates (CD, UC, IC, and HC).

The diversity of all potential novel species of gut microbial members (either from HC or disease-associated samples) based on taxonomic lineage is depicted in Figure 5. The cladogram (based on 16S rRNA gene sequences) contained 245 strains representing at least 67 different species and 44 genera from 27 families belonging to five phyla (*Firmicutes*, *Bacteroidetes*, *Actinobacteria*, *Proteobacteria*, and *Verrumicrobia*), which provided unprecedented microbial diversity in the human gut. The ratio of novel microbes isolated from disease-associated fecal samples to HCs was 8:53 (Supplementary Figure S21a). This ratio suggested that the microbes in the healthy gut are more diverse and comprise more novel uncultivated bacteria than in diseased conditions where the abundance of potential pathogenic microbes may elevated (Supplementary Tables S3, S5; Nishida et al., 2018). Moreover, among the potential novel species from both disease-associated samples and HCs, 134 bacterial strains were previously uncultured species. Each of the 134 strains exhibited top hits with uncultured bacterial clones in the NCBI standard database, which amounts to approximately 55% of novel isolates belonging to previously uncultured clones of human gut bacteria (Supplementary Table S7). The deposition of each novel bacterial strain in at least two culture banks in two different countries, characterization, and comprehensive analyses of all strains are under investigation. Further approaches are required to illustrate the functional aspects of novel gut microbiota and their relationship with gut health. Moreover, we isolated bacterial species, such as *Bacillus altitudinis*, *Bacillus piscis*, *Brevibacterium frigoritolerans*, *Microbacterium aoyamense*, *Microbacterium hydrothermale*, *Micrococcus antarcticus*, and *Priestia megaterium*, which have never been reported in the human gut. Additionally, we isolated *Streptococcus periodonticum* strains from healthy fecal samples which was originally isolated from human dental plaque (Lim et al., 2019) and have not been reported in the human gut.

### qPCR amplification

**Figure 6:**
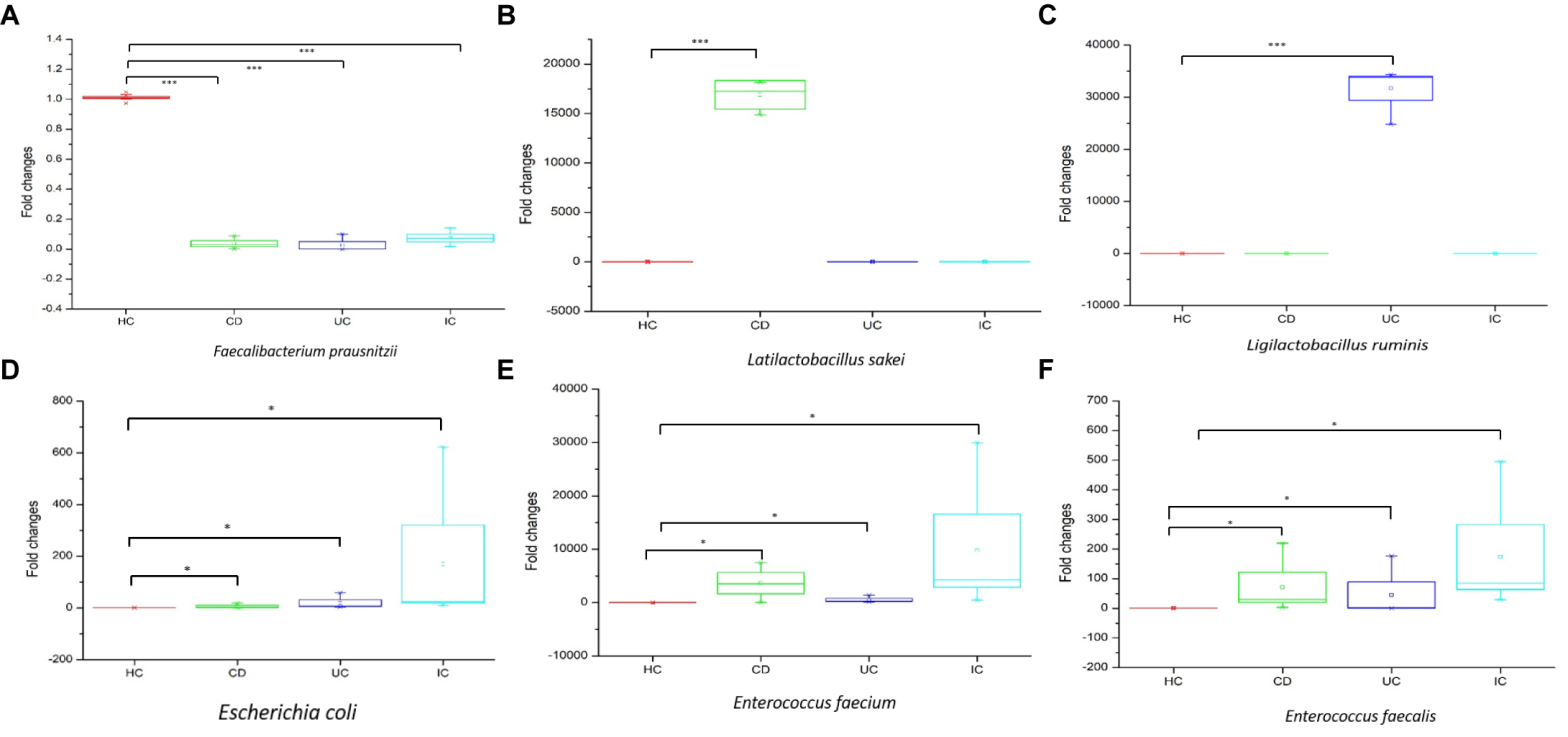
The qPCR analysis of upregulation and downregulation of 16S rRNA genes of specific species during CD, UC, and IC compared to healthy control (HC). Statistical significance was measured based on Wilcoxon rank-sum test. *p < 0.05; ***p < 0.001.

qPCR amplifications were performed to validate the upregulation or downregulation of specific bacterial genes during IBD or IC conditions compared with that in HCs. In all cases of IBD (CD and UC) or IC, the beneficial bacteria for human health, that is, *Faecalibacterium prausnitzii*, were significantly reduced compared with that in HCs (Figure 6A). We observed notable changes in *Latilactobacillus sakei* in CD and *Ligilactobacillus ruminis* in UC. In both cases, the 16S rRNA gene copy number was significantly increased compared with that in the healthy control (Figures 6B,C). Notably, the 16S rRNA gene copy numbers of potential pathogenic species such as *Escherichia coli*, *Enterococcus faecium*, and *Enterococcus faecalis* increased under IC conditions (Figures 6D–F).

## Discussion

**Figure 7:**
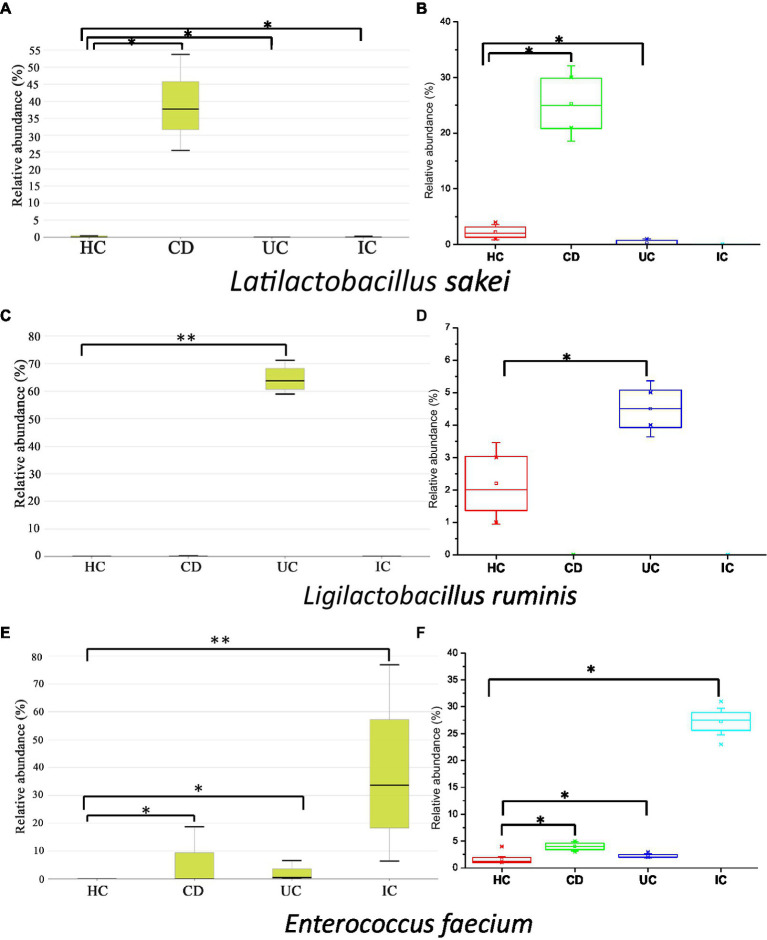
Relative abundance of *Latilactobacillus sakei* **(A,B)**, *Ligilactobacillus ruminis* **(C,D)**, and *Enterococcus faecium* **(E,F)** in CD, UC, IC, and HC conditions. Data were from 16-based MTP **(A,C,E)** and culturomics **(B,D,F)**. Statistical significance was measured based on Wilcoxon rank-sum test. *p < 0.05; **p < 0.01.

In this study, we observed the phylum-to-species-level gut microbial abundance in disease-associated as well as healthy individual fecal samples from both (culture- and non-culture-based) approaches. We demonstrated that the abundance of multiple orders, classes, families, genera, and species differed between IBD conditions (CD and UC), IBD and IC, and each IBD or IC condition and HC. An increase in the relative abundance of *Firmicutes* and a decrease in the level of *Bacteroidetes* were observed in all disease-associated (CD, UC, or IC) samples (Figure 1). Similar changes in gut microbiota during IBD have been reported in previous studies (Alam et al., 2020). Notably, for CD patients, the abundance levels of five classes (*Bacilli*, *Clostridia*, *Actinobacteria*, *Erysipelotrichia*, and *Coriobacteriia*) (Supplementary Figure S4b), five families (*Lactobacillaceae*, *Peptostreptococcaceae*, *Streptococcaceae*, *Enterococcaceae*, and *Erysipelotrichaceae*) (Supplementary Figure S5b), five genera (*Lactobacillus*, *Romboutsia*, *Enterococcus*, *Streptococcus*, and *Eggerthella*) (Supplementary Figure S8), and five species (*Latilactobacillus sakei*, *Romboutsia timonensis*, *Enterococcus faecium*, *Clostridium innocuum*, and *Intestinibacter bartlettii*) (Supplementary Figure S11) were increased. Similarly, in patients with UC, the abundance levels of four classes (*Bacilli*, *Clostridia*, *Actinobacteria*, and *Erysipelotrichia*; Supplementary Figure S4b), four families (*Lactobacillaceae*, *Peptostreptococcaceae*, *Streptococcaceae*, and *Enterococcaceae*) (Supplementary Figure S5b), four genera (*Lactobacillus*, *Streptococcus*, *Enterococcus*, and *Intestinibacter*) (Supplementary Figure S8), and four species (*Ligilactobacillus ruminis*, *Streptococcus salivarius*, *Enterococcus faecium*, and *Intestinibacter bartlettii*) (Supplementary Figure S11) were elevated. Additionally, investigating species such as *Bacillus paralicheniformis*, *Eggerthella lenta*, *Eggerthella sinensis*, *Alistipes finegoldii, Catabacter hongkongensis,* and *Eubacterium callanderi*, which have been isolated from fecal samples associated with IBD would be prudent. Moreover, we isolated multiple *Kalameilla piersonii* strains that were not observed in the 16S-based MTP profile (NGS microbiome data). A previous study reported that *K. piersonii* isolated from urine plays a vital role in struvite crystallization (Rekha et al., 2020). This species would also be of interest in future studies on IBD dysbiosis. Notably, the abundance of *Latilactobacillus sakei* and *Enterococcus faecium* in CD conditions, as well as *Ligilactobacillus ruminis* and *Enterococcus faecium* under UC conditions, were increased when the data were analyzed by both (non-culture and culture-based) approaches (Figure 7 and Supplementary Figure S22) and this was further validated by qPCR data (Figure 6). Hence, our data suggest that the decreased abundance of *Faecalibacterium prausnitzii* and increased abundance of *Latilactobacillus sakei,* and *Enterococcus faecium* could be biomarkers of CD. Similarly, decreased abundance of *Faecalibacterium prausnitzii* and an increased abundance of *Ligilactobacillus ruminis* and *Enterococcus faecium* could be biomarkers for UC. Finally, an increased abundance of *Enterococcus faecium* and *Escherichia coli* and a decreased abundance of *Faecalibacterium prausnitzii* could be markers for IC (Figures 6, 7 and Supplementary Figure S22). The decreased abundance (in fold changes) of *Faecalibacterium prausnitzii* under diseased conditions was further validated by qPCR (Figure 6A). A comparative method of relative quantification (by the ΔΔC_T_ method) of the 16S rRNA gene suggested that the abundance of *Faecalibacterium prausnitzii* during disease was much more reduced than that of healthy individuals (Figure 6A). The highly increased abundance of *Latilactobacillus sakei* during CD and *Ligilactobacillus ruminis* during UC was also supported by gene quantification (Figures 6B,C). Moreover, the increased *Enterococcus faecium*, *Escherichia coli*, and *Enterococcus faecalis* abundance during IC conditions was supported by qPCR data, and these three bacteria could be a marker for diagnosing IC (Figures 6, 7 and Supplementary Figure S22).

Gut dysbiosis is associated with disease phenotypes, and the abundance of particular species (diversity of gut microbiota) depends on geographical location, ethnicity, diet, and lifestyle (Gupta et al., 2017; Almeida et al., 2019). Therefore, comprehensively investigating these species to confirm their disease-inducing abilities would be promising. *Faecalibacterium prausnitzii*, regarded as a vital marker of a healthy gut, produces butyrate (short-chain fatty acid, SCFA) in the gut, which has protective properties against colon inflammation (Laursen et al., 2017). The depletion of this species from the gut may indicate inflammatory diseases such as IBD, IC, RA, and CRC (Lopez-Siles et al., 2017).

For the culturomics, 2,238 pure cultures were obtained from all 21 fecal samples consisting of six phyla, 13 classes, 27 orders, 51 families, 110 genera, and 241 species. The total number of isolates from each sample type is depicted in Supplementary Figure S21. All the 16S rRNA gene sequences of the 2,238 strains have already been deposited in the NCBI database with available GenBank accession numbers (Supplementary Tables S3, S5). The human gut microbiota possesses a diverse bacterial community directly associated with human health and disease (Browne et al., 2016). Although several attempts have been made to culture previously uncultured species from the human gut, the vast majority of human gut bacteria have not been cultured yet (Pfleiderer et al., 2013; Browne et al., 2016; Lau et al., 2016; Das et al., 2018; Liu et al., 2021). We explored numerous previously uncultured species from the human gut microbiome, and further study of these novel species will provide insight into the gut microbiome and will resolve the taxonomic resolution of gut microbiota to some extent. Additionally, the newly isolated species will provide an understanding of microbiome functions in the human gut and provide functional novelty. Moreover, we identified 40 potential novel probiotic strains based on phylogenetic analysis of 25 potential novel species that might have beneficial health effects, including enhancement of the host immune response and antimicrobial, anticancer, anti-inflammatory, and antiallergic properties (Supplementary Table S8; Degruttola et al., 2016; Bazireh et al., 2020; Fong et al., 2020). Probiotics are capable of restoring the impaired microbiome of a dysbiotic gut. They are, therefore, highly valuable in maintaining the dysbiosis caused by IBD, IC, colorectal cancer (CRC), IBS, obesity, diabetes mellitus (type 1), allergic disorders, and autism (Degruttola et al., 2016; Fong et al., 2020). Various studies suggest that probiotics for human use originate from humans (Bazireh et al., 2020). We isolated potential probiotic strains from fecal samples of healthy individuals that met the criteria to define probiotics. And, further studies involving numerous morphological and biochemical tests, as well as probiotic efficacy and safety, are required and are currently under investigation.

## Conclusion

We performed the extensive study of microbial imbalances in IBD/IC patients at different taxonomic levels both by culture-independent and culturomic approaches and our study demonstrated that species richness in all disease-associated (CD, UC, or IC) conditions was reduced, and imbalances in phyla, order classes, families, genera, and species were observed. From both (non-culture and culture-based) perspectives, the highly increased abundance of *Latilactobacillus sakei* and *Enterococcus faecium* in CD, *Ligilactobacillus ruminis* and *Enterococcus faecium* in UC, and *Enterococcus faecium*, *Escherichia coli*, and *Enterococcus faecalis* in IC could be biomarkers for CD, UC, and IC, respectively. These results confirm taxonomic and functional differences in the gut microbiota of CD, UC, or IC patients compared with that in healthy individuals. Additionally, 2,238 isolates of human gut microbiota were isolated from 21 fecal samples of disease-associated (IBD and IC) or healthy Korean individuals comprising 245 strains of potentially novel bacterial isolates from 27 families, including 15 potential novel genera and at least 67 potential novel species. Of the 245 isolates, 134 were previously uncultured bacterial clones from the human gut microbiota. We expect that the exploration of these novel species from human fecal samples provides extensive taxonomic resolution and diversity of human gut microbiota and its association with human health, as well as elucidates the mechanism of host–microbiota interaction. As the source of the sample (country-specific differences in gut microbiota owing to a specific diet or environmental factors) could be a critical factor in exploring more novel bacteria from Korean individuals, fresh samples of additional volunteers are required to explore the entire perspective of the Korean gut microbiota.

## Data availability statement

The datasets presented in this study can be found in online repositories. The names of the repository/repositories and accession number(s) can be found in the article/Supplementary material.

## Ethics statement

The studies involving human participants were reviewed and approved by Institutional Review Board of Kyungpook National University Hospital (KNUH 2021-03-011-002). The patients/participants provided their written informed consent to participate in this study.

## Author contributions

RD conceived, designed, and conducted all the experiments. SK, YK, and EK interpreted the data. JK coordinated and supervised this study. RD, SK, and JK analyzed the data and prepared the manuscript. All authors discussed the results and contributed to the final manuscript.

## Funding

This research was supported by the Basic Science Research Program through the National Research Foundation of Korea (NRF) funded by the Ministry of Education (grant number NRF-2017R1D1A3B06032486).

## Conflict of interest

The authors declare that the research was conducted in the absence of any commercial or financial relationships that could be construed as a potential conflict of interest.

## Publisher’s note

All claims expressed in this article are solely those of the authors and do not necessarily represent those of their affiliated organizations, or those of the publisher, the editors and the reviewers. Any product that may be evaluated in this article, or claim that may be made by its manufacturer, is not guaranteed or endorsed by the publisher.

## Footnotes

^1^ https://www.ezbiocloud.net/identify

^2^ https://blast.ncbi.nlm.nih.gov/Blast.cgi?PAGE_TYPE=BlastSearch

^3^ https://www.ezbiocloud.net/contents/16smtp

^4^ https://mothur.org/wiki/rdp_reference_files

^5^ https://help.ezbiocloud.net/mtp-pipeline/

## Abbreviations

CD: Crohn’s disease
CRC: colorectal cancer
GI: gastrointestinal
HC: healthy control
IBD: inflammatory bowel disease
IC: ischemic colitis
MS: multiple sclerosis
MTP: microbiome taxonomic profiling
qPCR: quantitative real-time polymerase chain reaction
RA: rheumatoid arthritis
SCFA: short chain fatty acid
UC: ulcerative colitis
