# Supplementary material for: Insight into gut dysbiosis of patients with inflammatory bowel disease and ischemic colitis
Source: Front Microbiol. 2023 May 11;14:1174832. doi: 10.3389/fmicb.2023.1174832 (PMC10211348; doi:10.3389/fmicb.2023.1174832)
Supplement: Supplementary file 9 [file Data_Sheet_1.pdf]

Supplementary Material

Insight into gut dysbiosis of patients with inflammatory bowel disease and ischemic colitis

[1]Ram Hari Dahal, [1]Shukho Kim, [2]Yu Kyung Kim, [3]Eun Soo Kim, [1]Jungmin Kim*

[1]Department of Microbiology, School of Medicine, Kyungpook National University, Daegu 41944, Republic of Korea

[2]Department of Clinical Pathology, School of Medicine, Kyungpook National University, Daegu 41944, Republic of Korea

[3]Department of Internal Medicine, School of Medicine, Kyungpook National University, Daegu 41944, Republic of Korea

Article type

Original Research

Running title

Inflammatory bowel disease and ischemic colitis

Corresponding author*

Phone: +82-53-420-4840; Fax: +82-53-427-5664; E-mail: minkim@knu.ac.kr

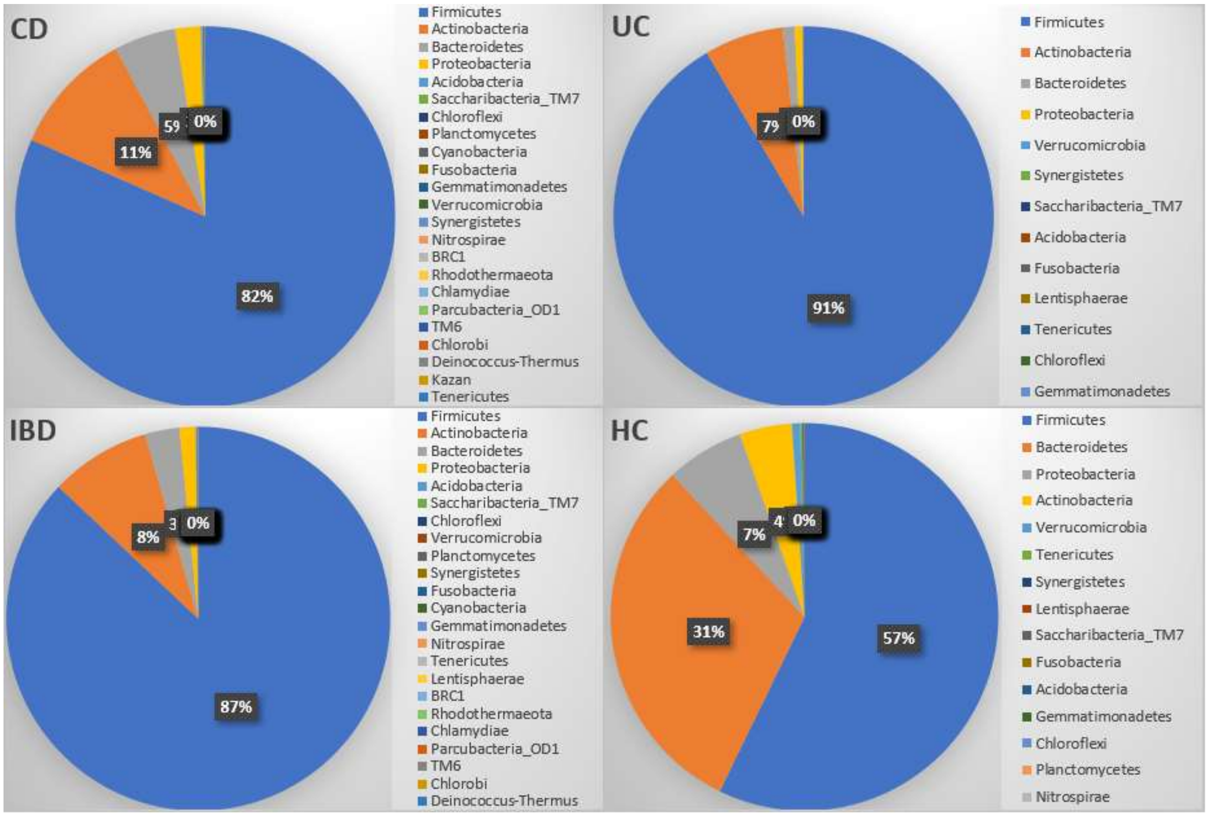

Figure S1 (a)

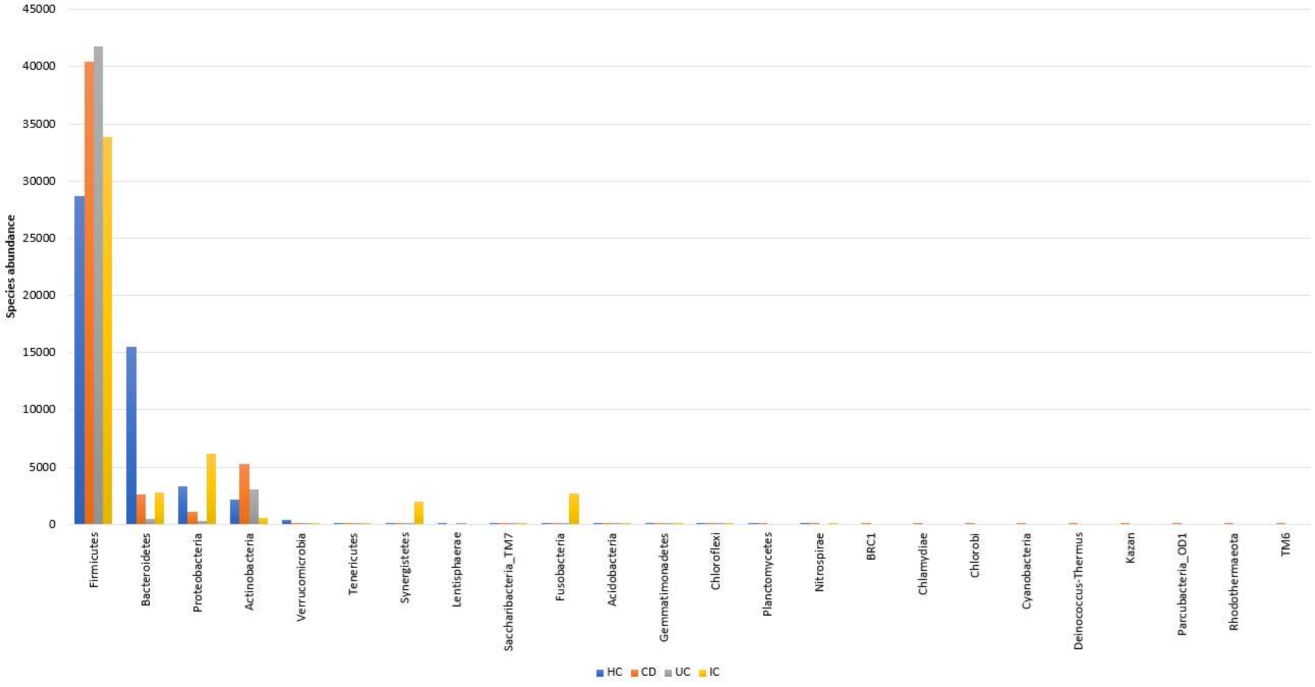

Figure S1 (b)

Supplementary Figure S1. Gut microbial abundance in different conditions at the phylum level. Phylum level abundance in CD, UC, IBD, and HC (a); HC, CD, UC, and IC (b).

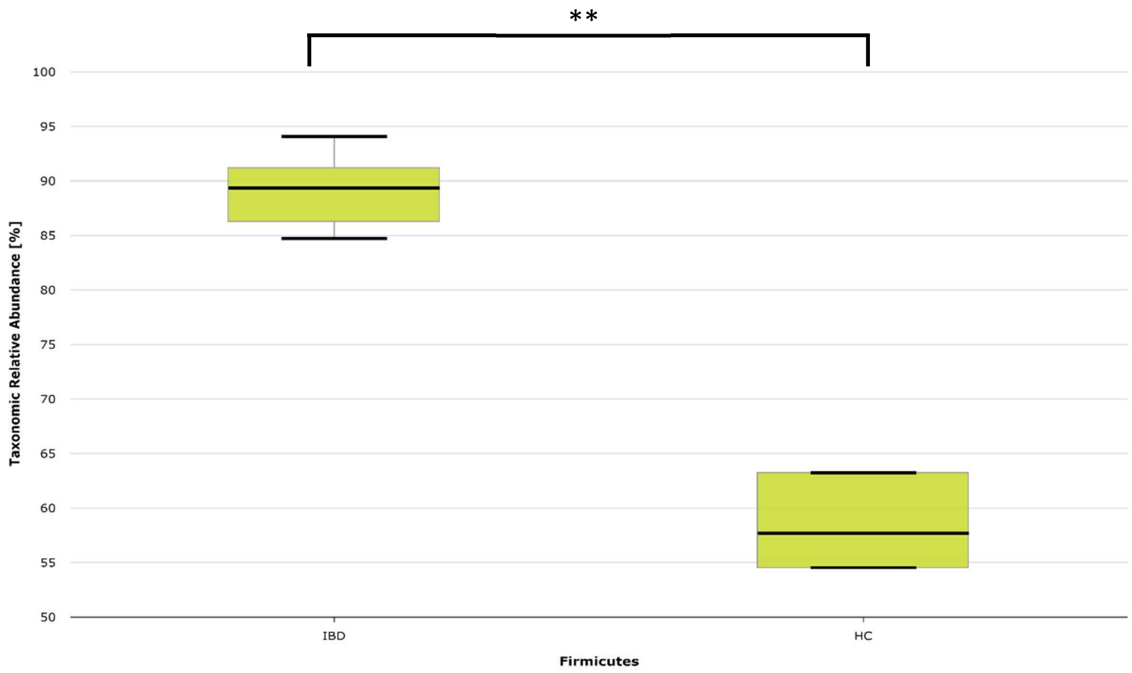

Supplementary Figure S2. Relative abundance of the phylum *Firmicutes* in IBD samples against healthy control. Statistical significance was measured based on Wilcoxon rank-sum test. **$P <$ 0.001.

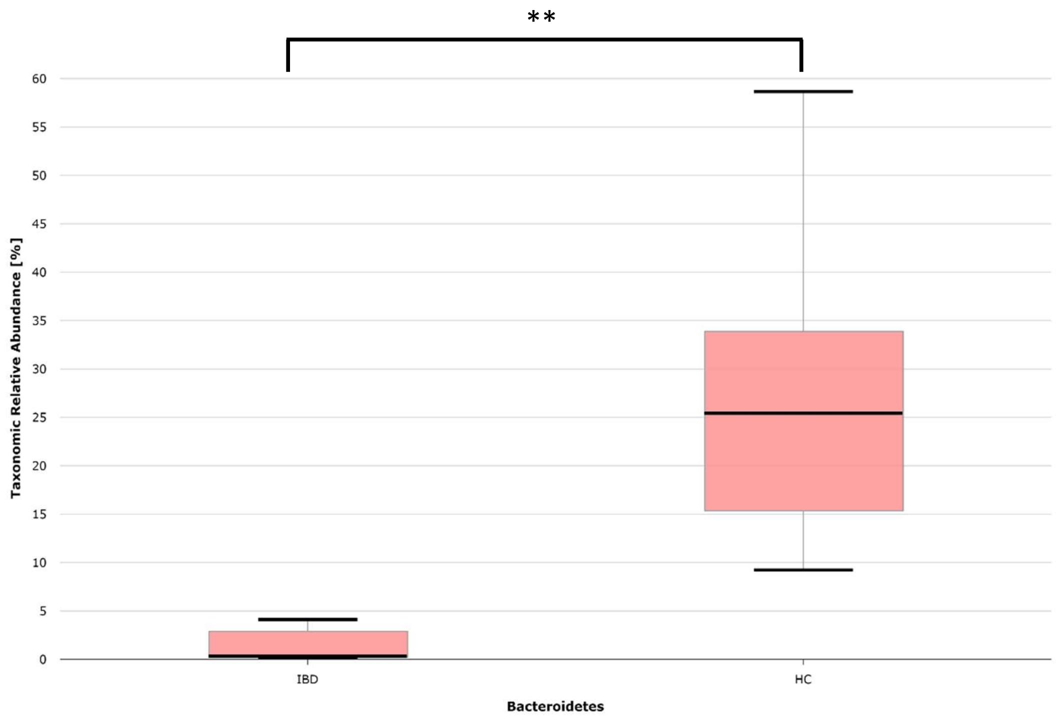

Supplementary Figure S3. Taxonomic relative abundance of the phylum *Bacteroidetes* in fecal samples of healthy subjects against IBD samples. Statistical significance was measured based on Wilcoxon rank-sum test. **$P < 0.001$.

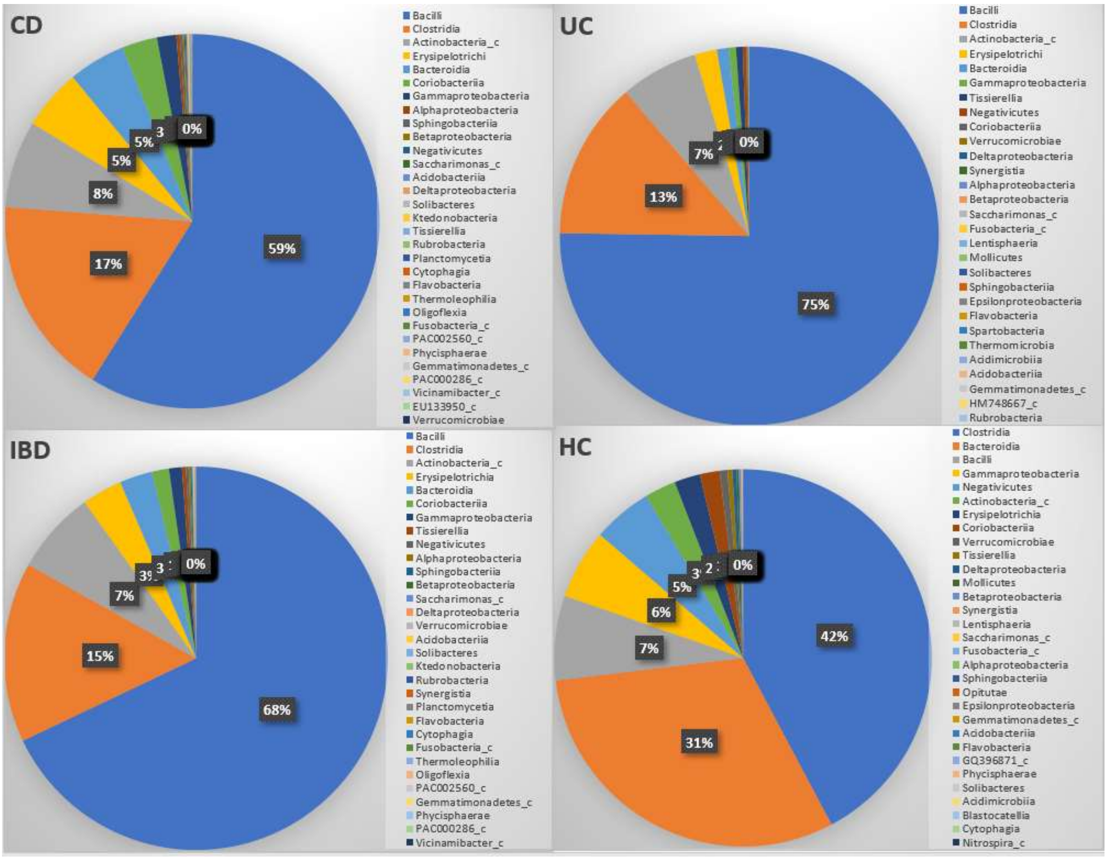

Figure S4 (a)

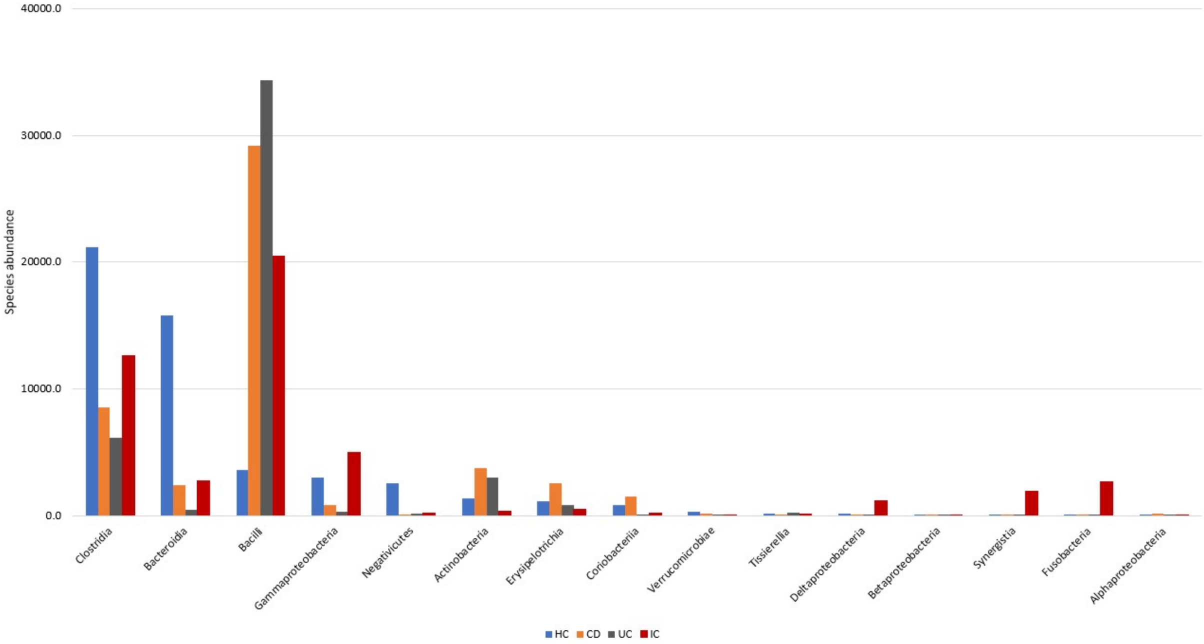

Figure S4 (b)

Supplementary Figure S4. Gut microbial abundance in different conditions at the class level. Class level abundance in CD, UC, IBD, and HC (a); HC, CD, UC, and IC (b).

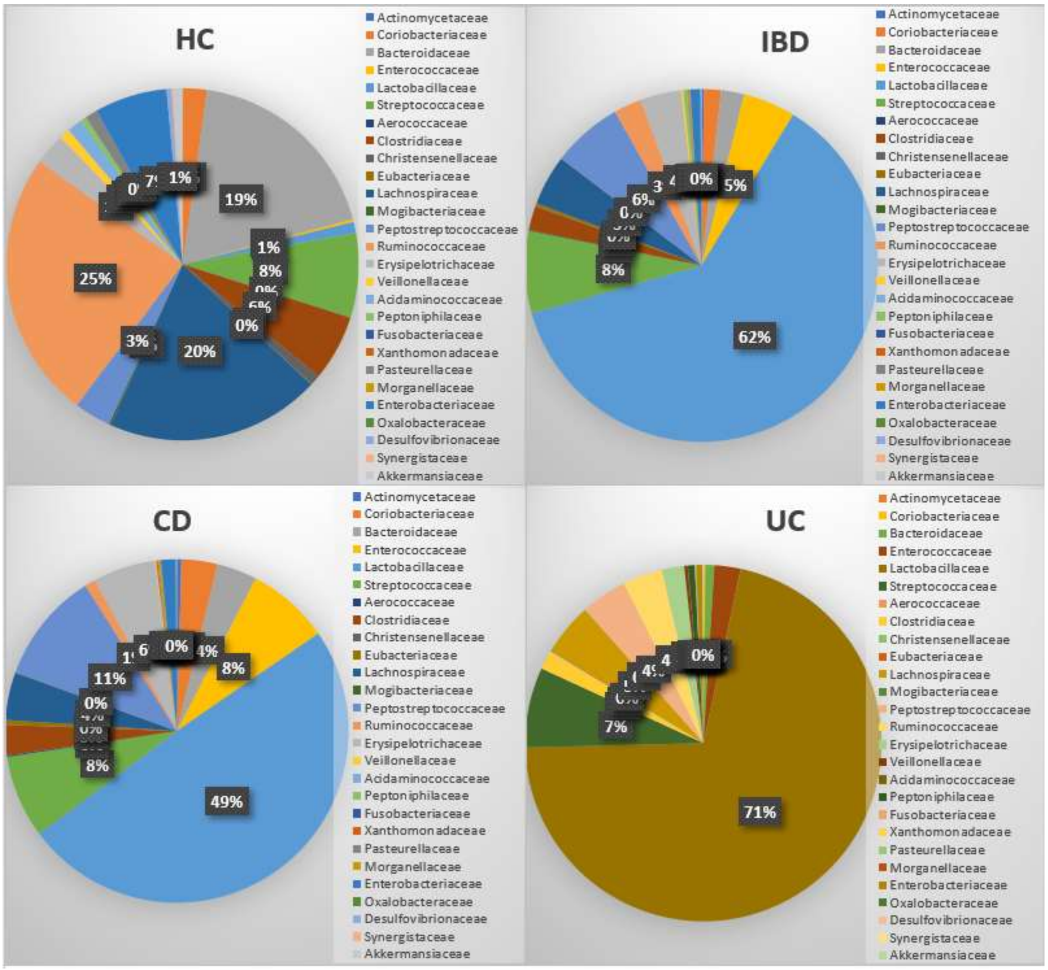

Figure S5 (a)

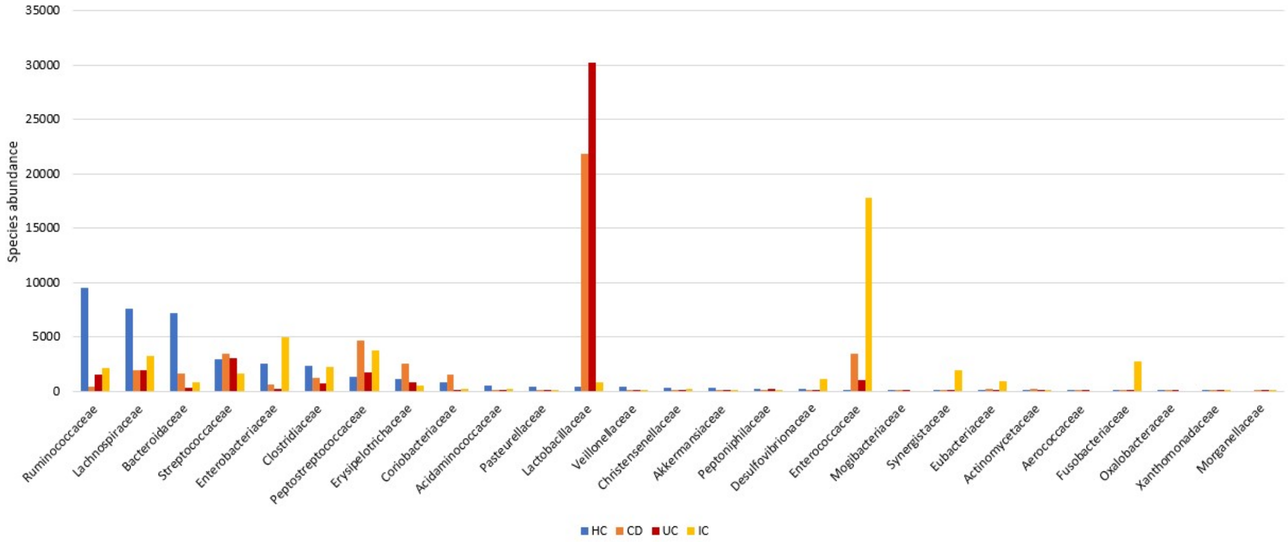

Figure S5 (b)

Supplementary Figure S5. Gut microbial abundance in different conditions at the family level. Family level abundance in HC, IBD, CD and UC (a); and HC, CD, UC and IC.

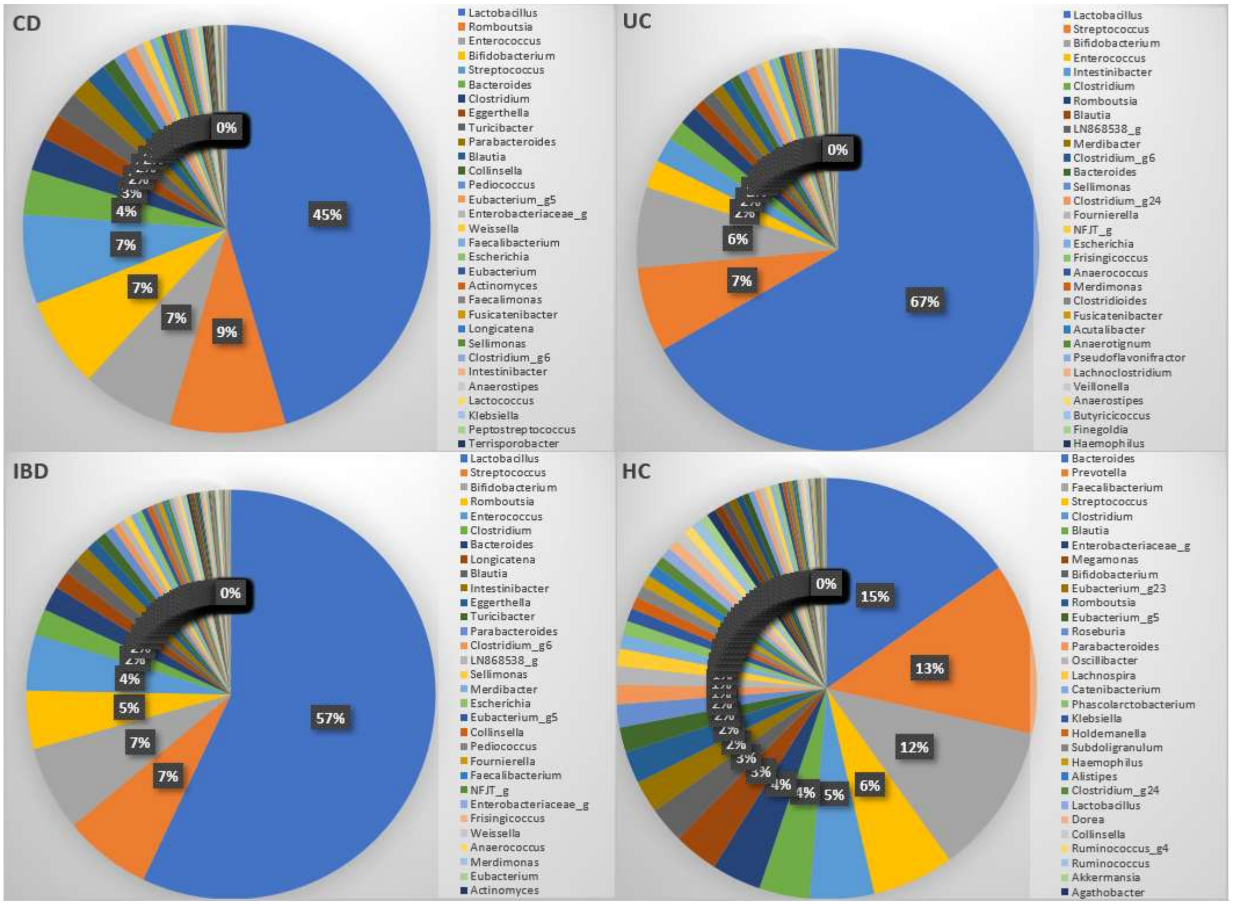

Figure S6 (a)

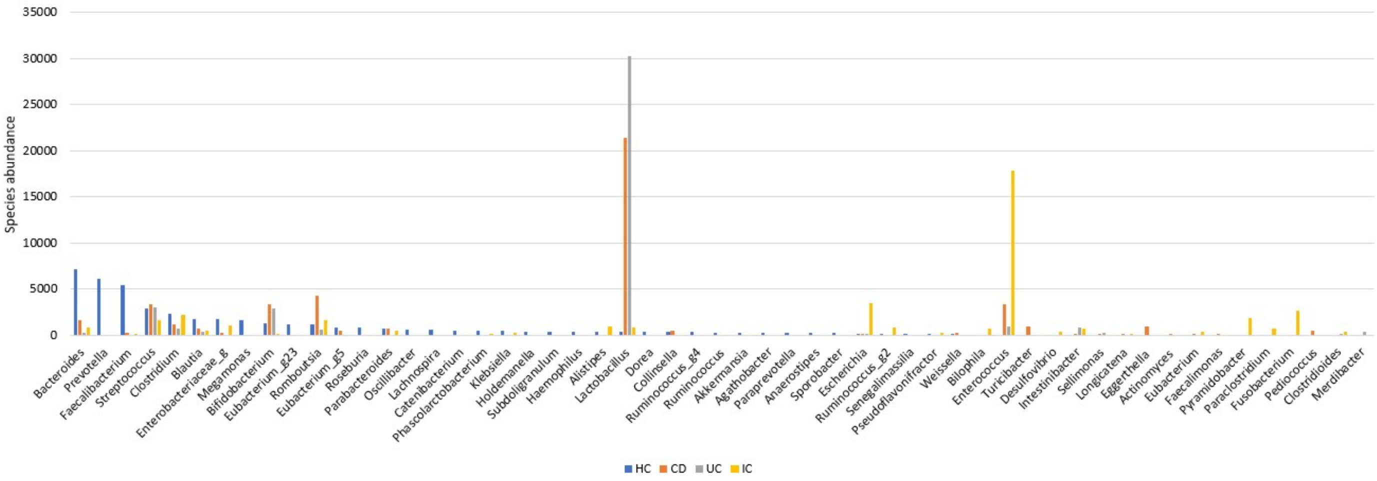

Figure S6 (b)

Supplementary Figure S6. Gut microbial abundance in different conditions at the genus level. Genus level abundance in CD, UC, IBD and HC (a); and HC, CD, UC, and IC

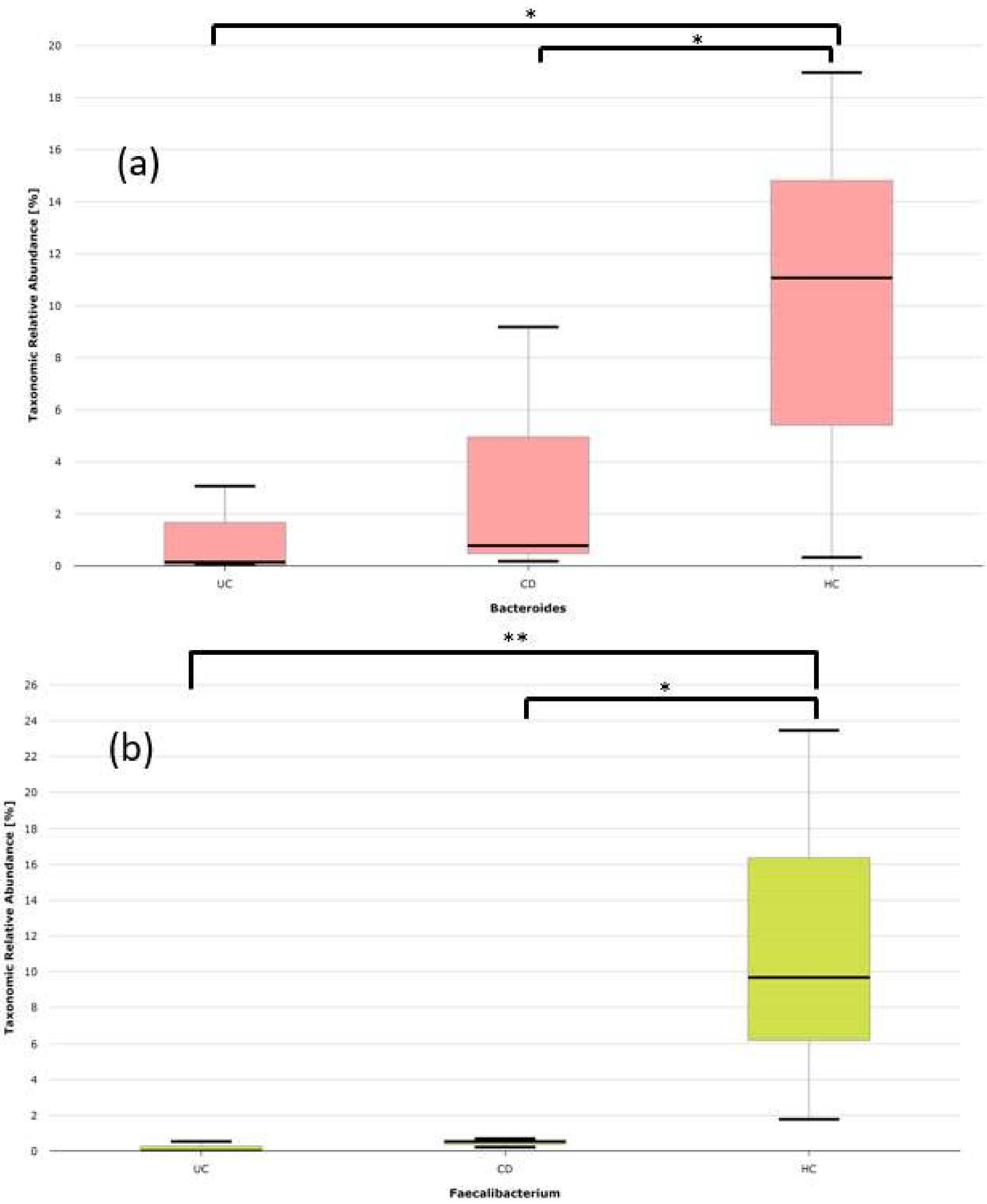

Supplementary Figure S7. Relative abundance of the genus *Bacteroides* (a) and *Faecalibacterium* (b) in fecal samples of healthy subjects against UC and CD. Statistical significance was measured based on Wilcoxon rank-sum test. $*P < 0.05$; $**P < 0.001$.

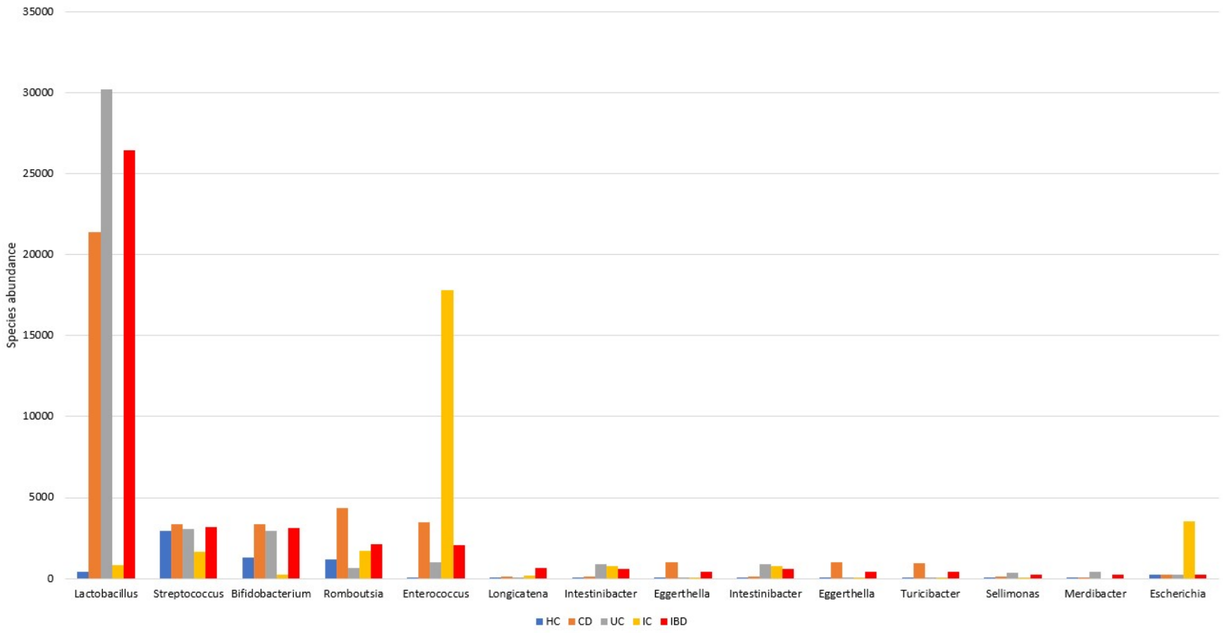

Supplementary Figure S8. Increased species abundance at the genus level during IBD and IC condition with respect to HC.

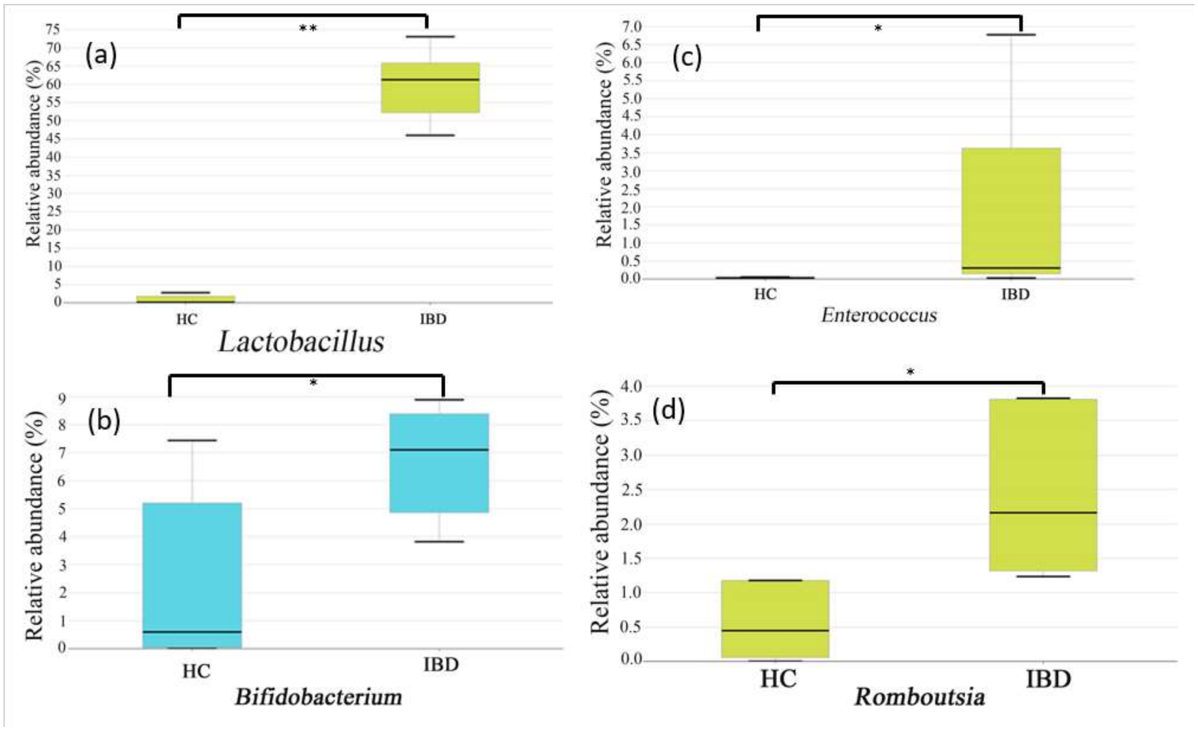

Supplementary Figure S9. Taxonomic relative abundance of the genus *Lactobacillus* (a), *Bifidobacterium* (b), *Enterococcus* (c), and *Romboutsia* (d) in fecal samples of healthy subjects against IBD. Statistical significance was measured based on Wilcoxon rank-sum test. $*P < 0.05$; $**P < 0.001$.

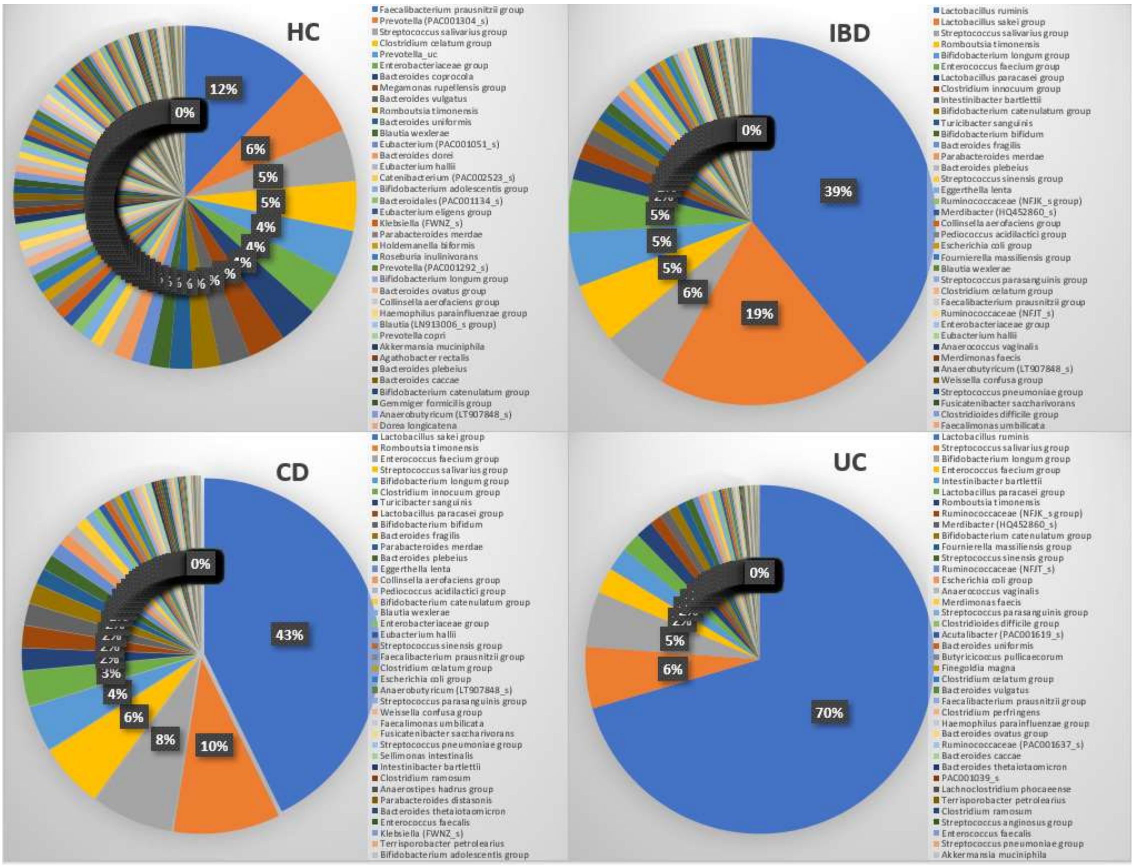

Figure S10 (a).

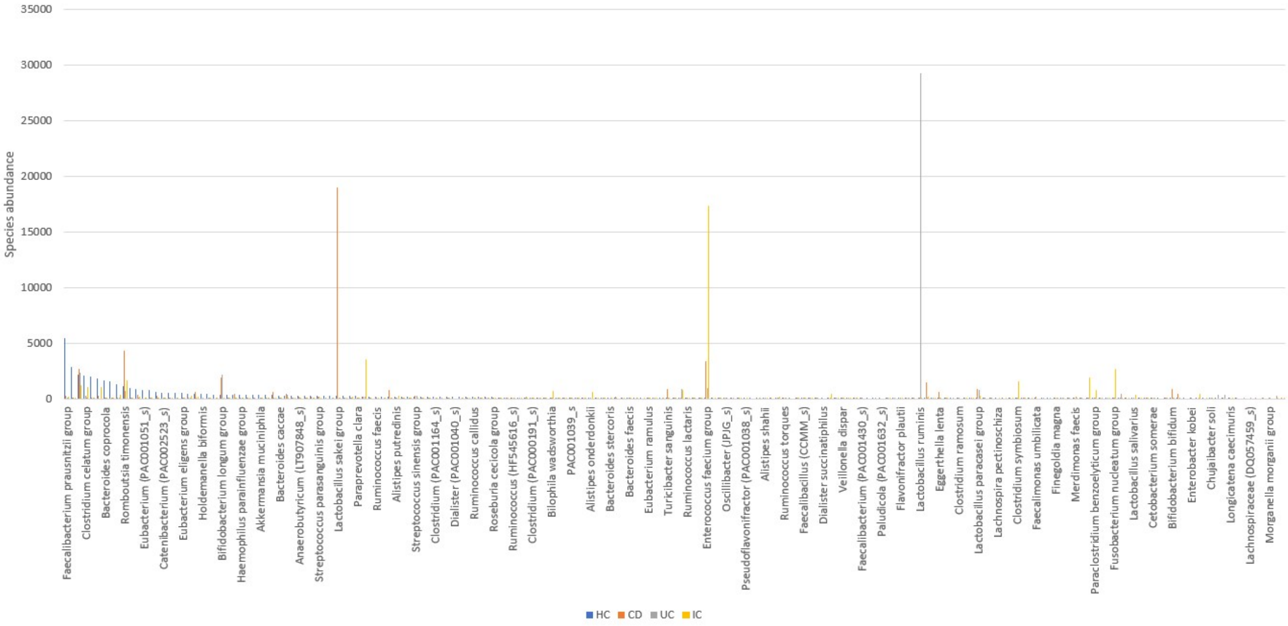

Figure S10 (b).

Supplementary Figure S10. Gut microbial abundance in different conditions at the species level. Species-level abundance in IBD, CD, UC IC, and HC (a); UC, CD, IC, and HC (b).

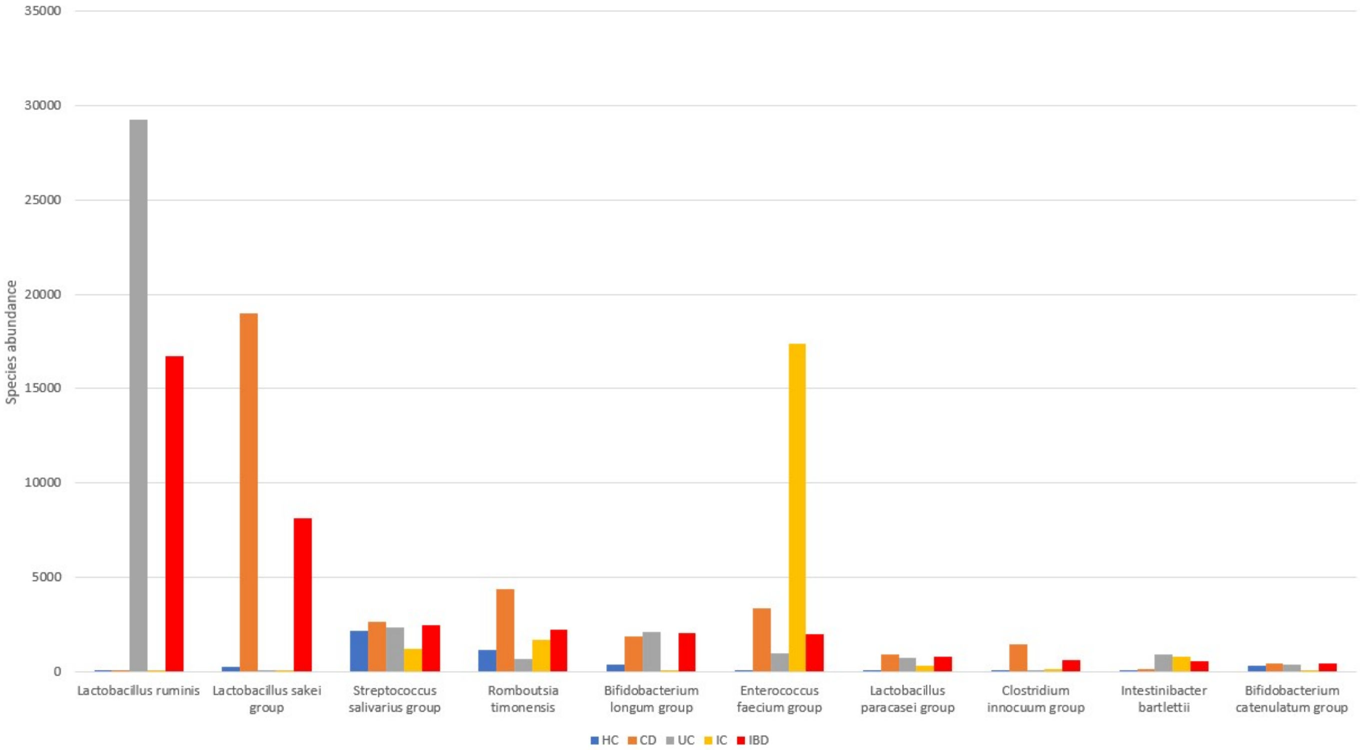

Supplementary Figure S11. Increased species abundance at the species level in HC, IBD and IC conditions.

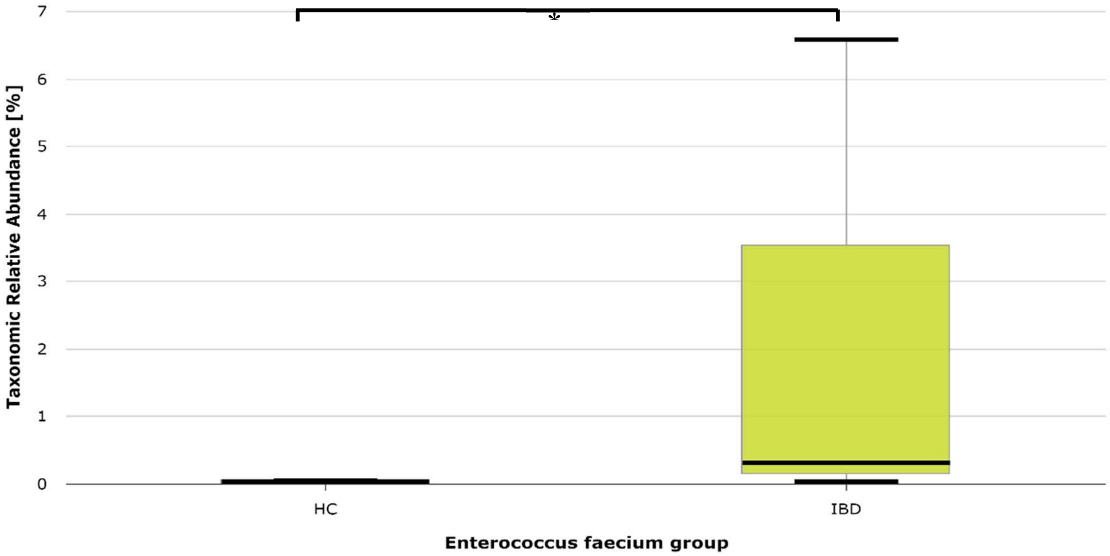

Supplementary Figure S12. Taxonomic relative abundance of *Enterococcus faecium* group in fecal samples of healthy subjects against IBD. Statistical significance was measured based on Wilcoxon rank-sum test. $^*P < 0.05$.

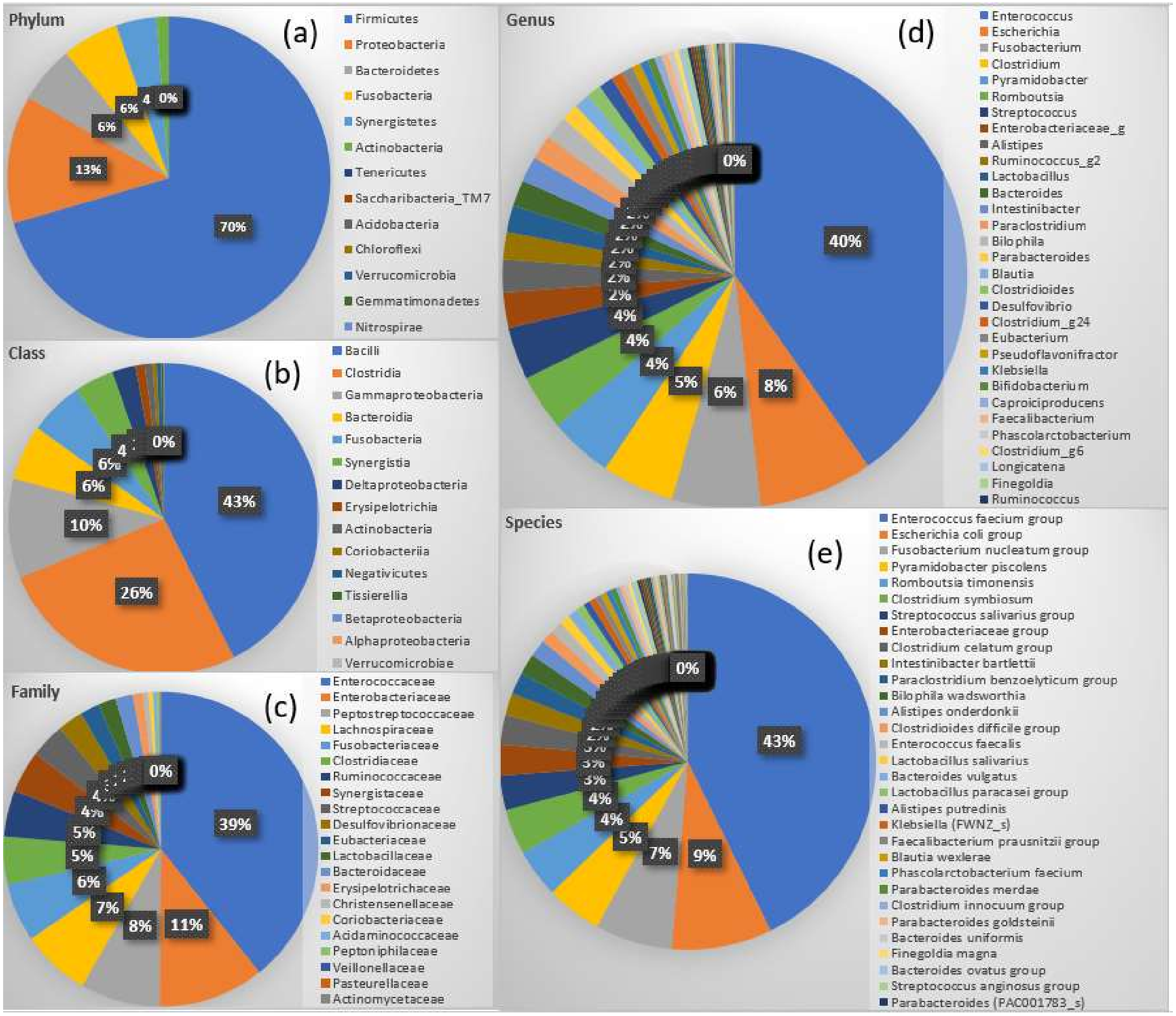

Supplementary Figure S13. The gut microbial abundance during IC. Abundance at phylum level (a); abundance at class level (b); abundance at family level (c); abundance at genus level (d); and abundance at species level (e).

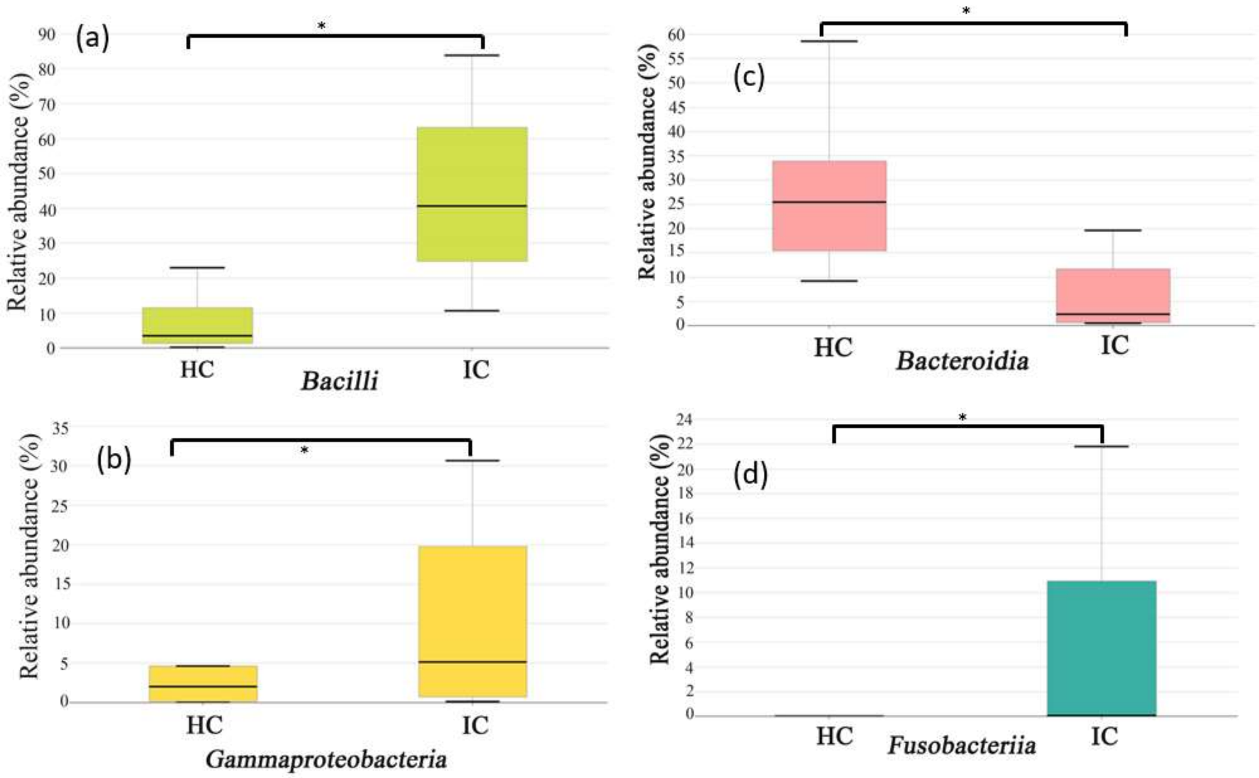

Supplementary Figure S14. Relative abundance at class level in faecal samples of IC against HC. *Bacilli* (a); *Gammaproteobacteria* (b); *Bacteroidia* (c); and *Fusobacteriia* (d). Statistical significance was measured based on Wilcoxon rank-sum test. $*P < 0.05$.

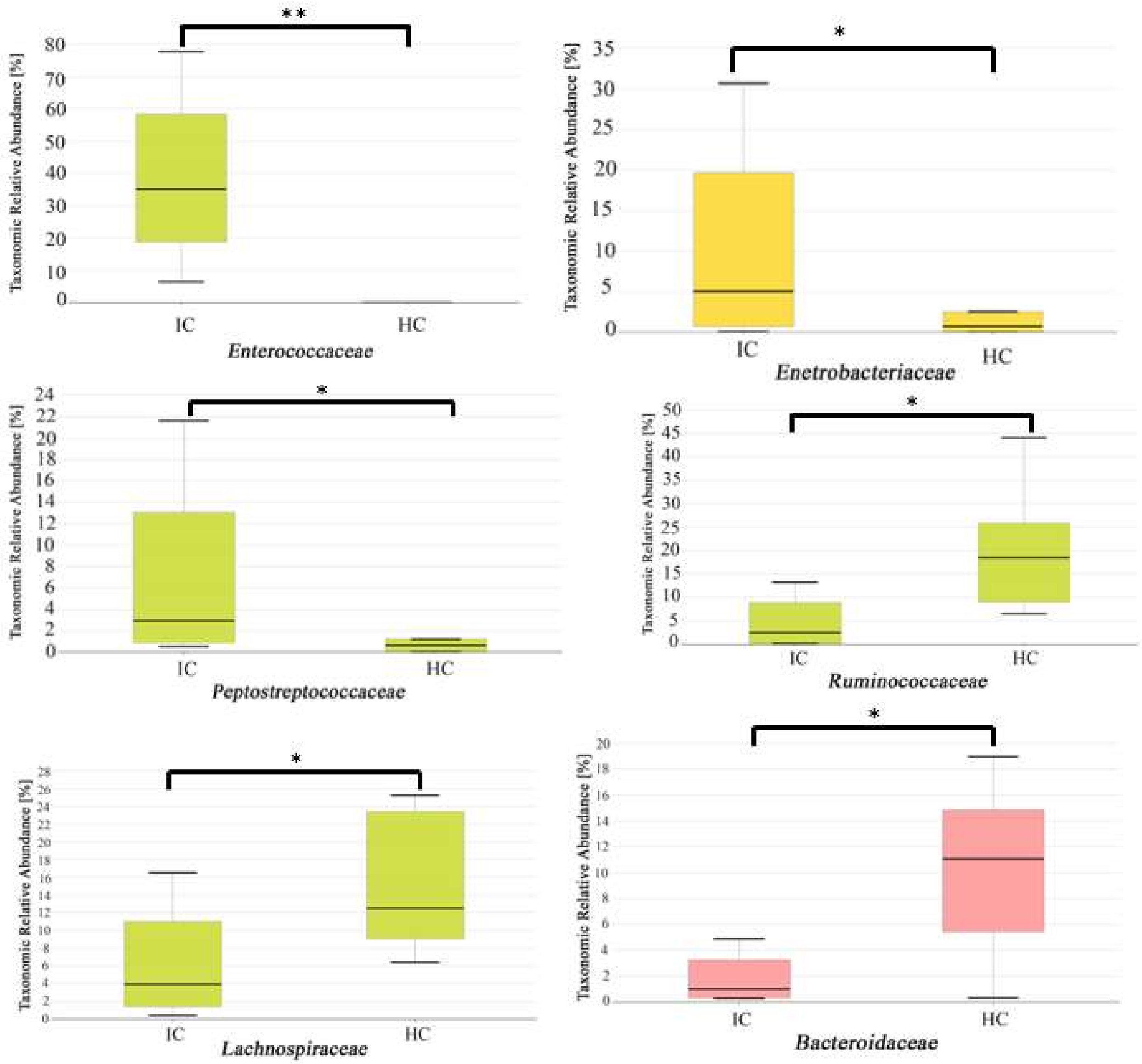

Supplementary Figure S15. Relative abundance at family level in faecal samples of IC against HC. Statistical significance was measured based on Wilcoxon rank-sum test. *$P < 0.05$; **$P < 0.001$.

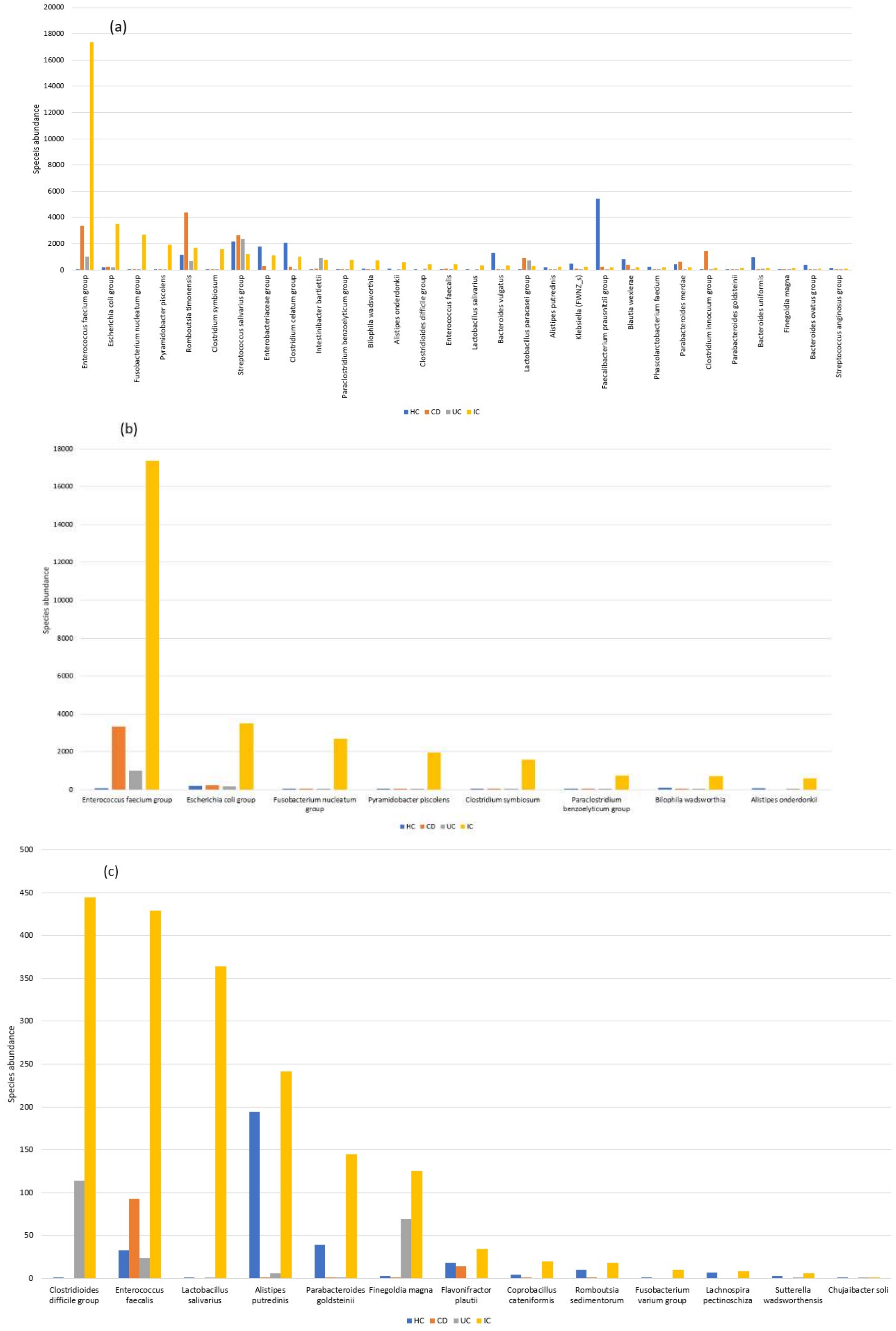

Supplementary Figure 16. Species level gut microbial abundance across various conditions. Figures (a, b, c) showed increased abundance during IC compared to HC, CD and UC.

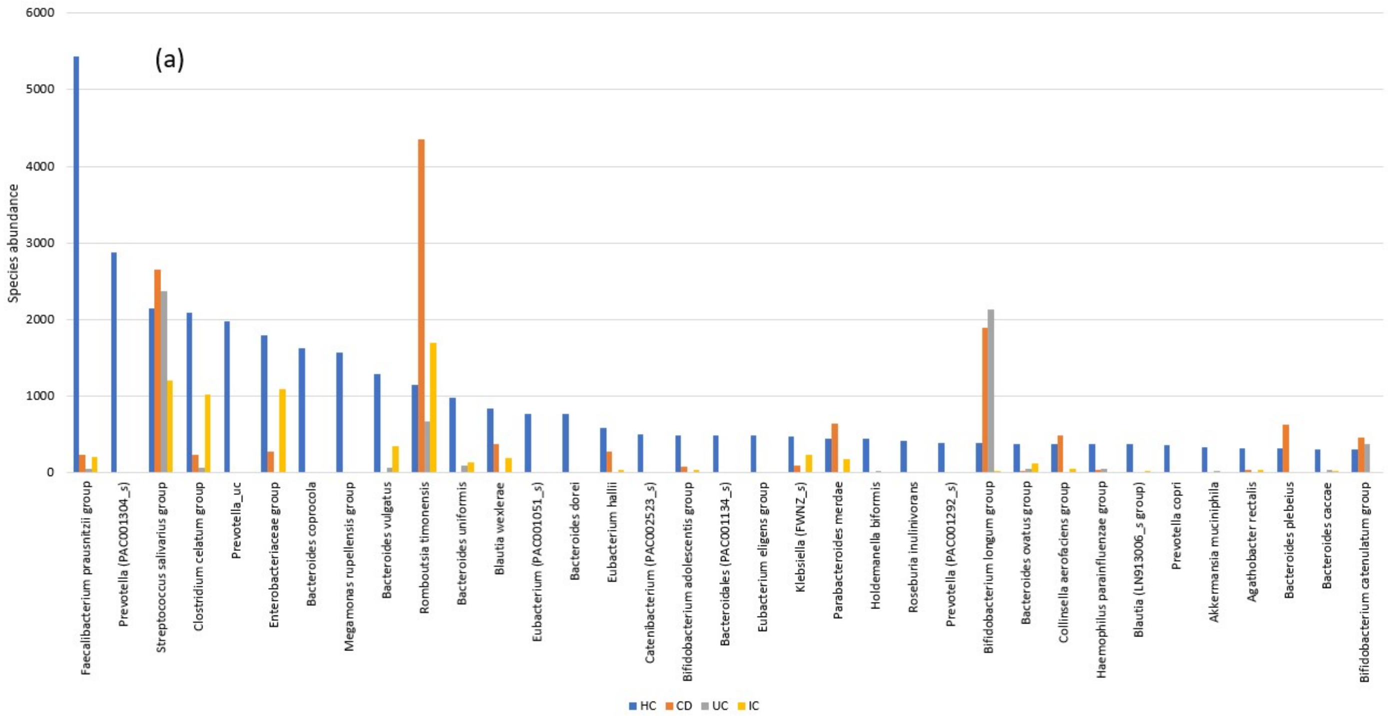
(a)
Species abundance
0
1000
2000
3000
4000
5000
6000
Faecalibacterium prausnitzii group
Prevotella (PAC001304_s)
Streptococcus salivarius group
Clostridium celatum group
Prevotella_uc
Enterobacteriaceae group
Bacteroides coprocola
Megamonas rupellensis group
Bacteroides vulgatus
Romboutsia timonensis
Bacteroides uniformis
Blautia wexlerae
Eubacterium (PAC001051_s)
Bacteroides dorei
Eubacterium hallii
Catenibacterium (PAC002523_s)
Bifidobacterium adolescentis group
Bacteroidales (PAC001134_s)
Eubacterium eligens group
Klebsiella (FWNZ_s)
Parabacteroides merdae
Holdemanella biformis
Roseburia inulinivorans
Prevotella (PAC001292_s)
Bifidobacterium longum group
Bacteroides ovatus group
Collinsella aerofaciens group
Haemophilus parainfluenzae group
Blautia (LN913006_s group)
Prevotella copri
Akkermansia muciniphila
Agathobacter rectalis
Bacteroides plebeius
Bacteroides caccae
Bifidobacterium catenulatum group
HC CD UC IC

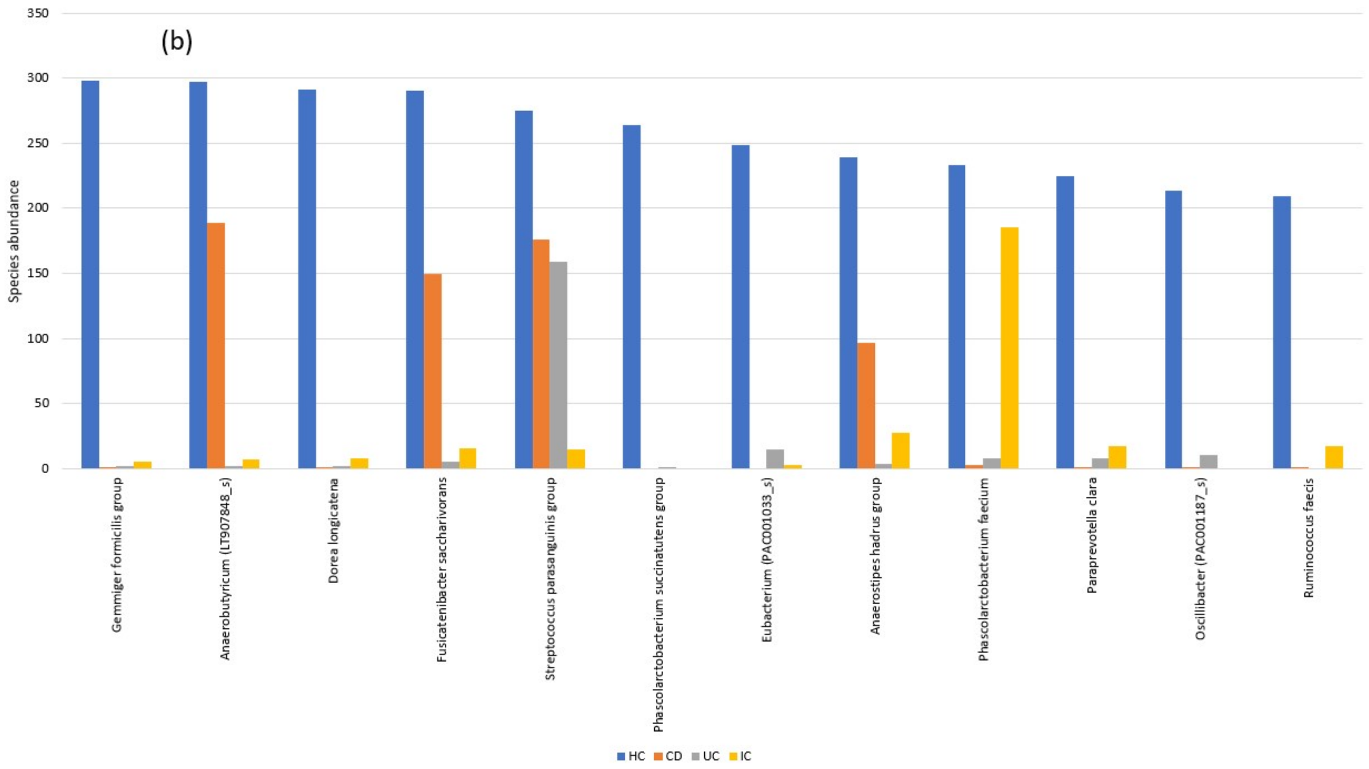
(b)
Species abundance
0
50
100
150
200
250
300
350
Gemmiger formicilis group
Anaerobutyricum (LT907848_s)
Dorea longicatena
Fusicatenibacter saccharivorans
Streptococcus parasanguinis group
Phascolarctobacterium succinatutens group
Eubacterium (PAC001033_s)
Anaerostipes hadrus group
Phascolarctobacterium faecium
Paraprevotella clara
Oscillibacter (PAC001187_s)
Ruminococcus faecis
HC CD UC IC

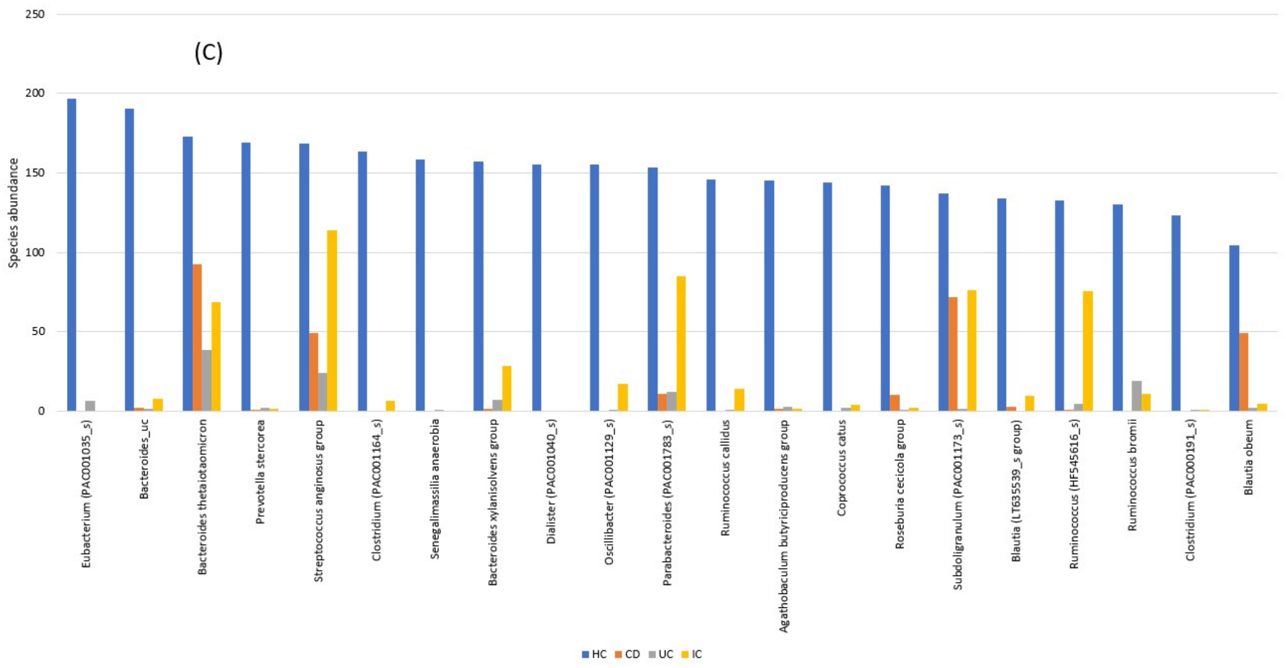
(C)
Species abundance
250
200
150
100
50
0
Eubacterium (PAC001035_s)
Bacteroides_uc
Bacteroides thetaiotaomicron
Prevotella stercorea
Streptococcus anginosus group
Clostridium (PAC001164_s)
Senegalimassilia anaerobia
Bacteroides xylanisolvens group
Dialister (PAC001040_s)
Oscillibacter (PAC001129_s)
Parabacteroides (PAC001783_s)
Ruminococcus callidus
Agathobaculum butyriciproducens group
Coprococcus catus
Roseburia cecicola group
Subdoligranulum (PAC001173_s)
Blautia (LT635539_s group)
Ruminococcus (HF545616_s)
Ruminococcus bromii
Clostridium (PAC000191_s)
Blautia obeum
HC CD UC IC

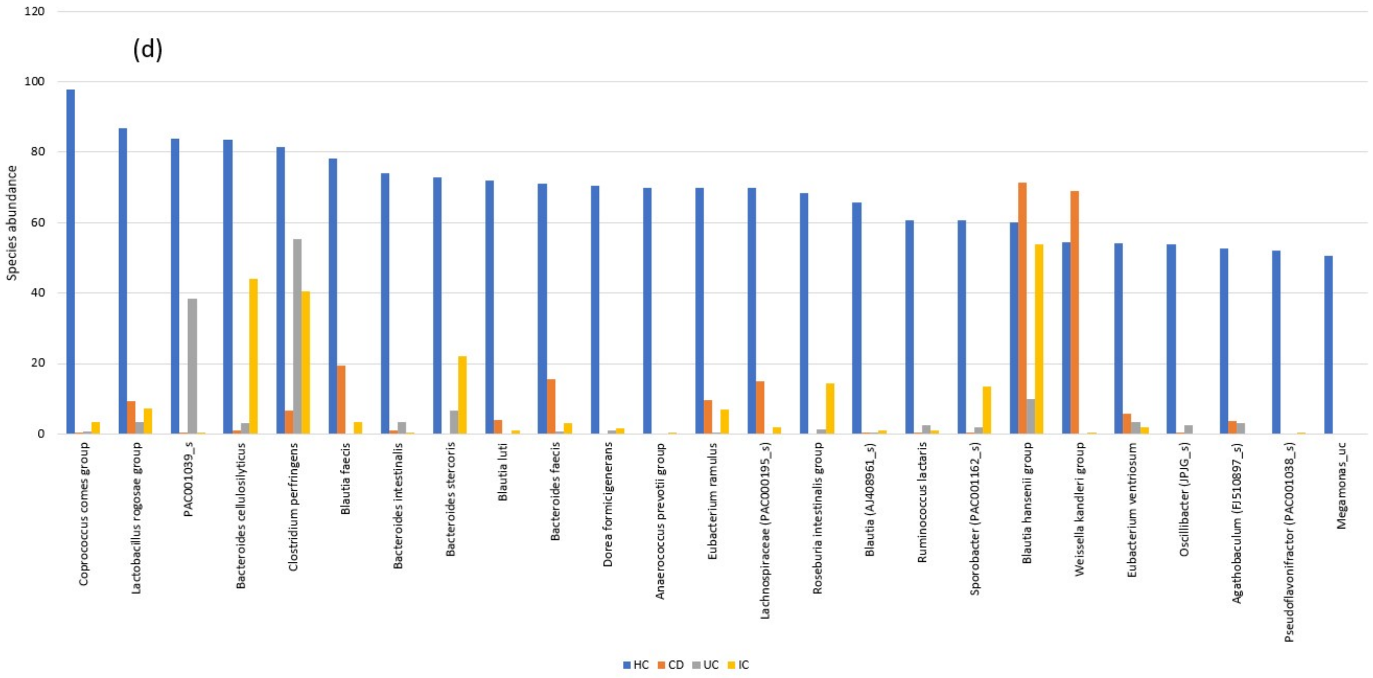
(d)
Species abundance
120
100
80
60
40
20
0
Coprococcus comes group
Lactobacillus rogosae group
PAC001039_s
Bacteroides cellulosilyticus
Clostridium perfringens
Blautia faecis
Bacteroides intestinalis
Bacteroides stercoris
Blautia luti
Bacteroides faecis
Dorea formicigenerans
Anaerococcus prevotii group
Eubacterium ramulus
Lachnospiraceae (PAC000195_s)
Roseburia intestinalis group
Blautia (AJ408961_s)
Ruminococcus lactaris
Sporobacter (PAC001162_s)
Blautia hansenii group
Weissella kandleri group
Eubacterium ventriosum
Oscillibacter (JPJG_s)
Agathobaculum (FJ510897_s)
Pseudoflavonifractor (PAC001038_s)
Megamonas_uc
HC CD UC IC

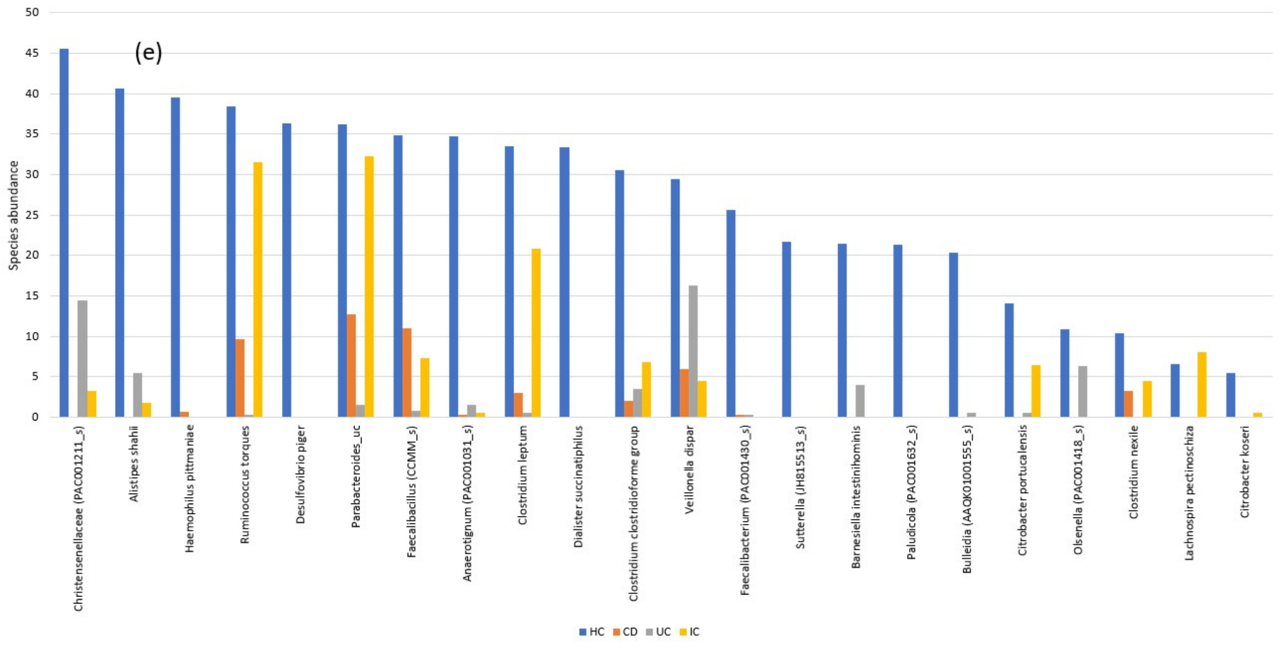

Supplementary Figure S17. Species level gut microbial abundance across various conditions. The figures (a, b, c, d, and e) showed higher abundance in HC compared to CD, UC and IC.

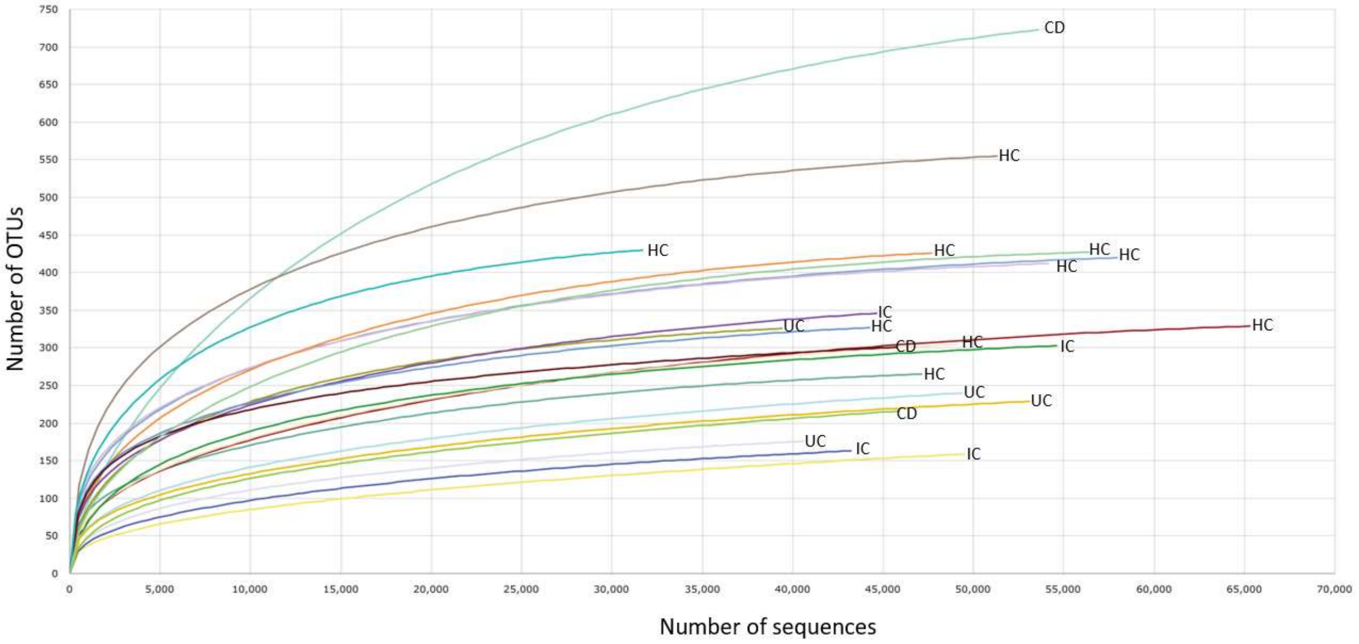

Supplementary Figure S18. The rarefaction curve of number of reads observed in HC, CD, UC, IC samples.

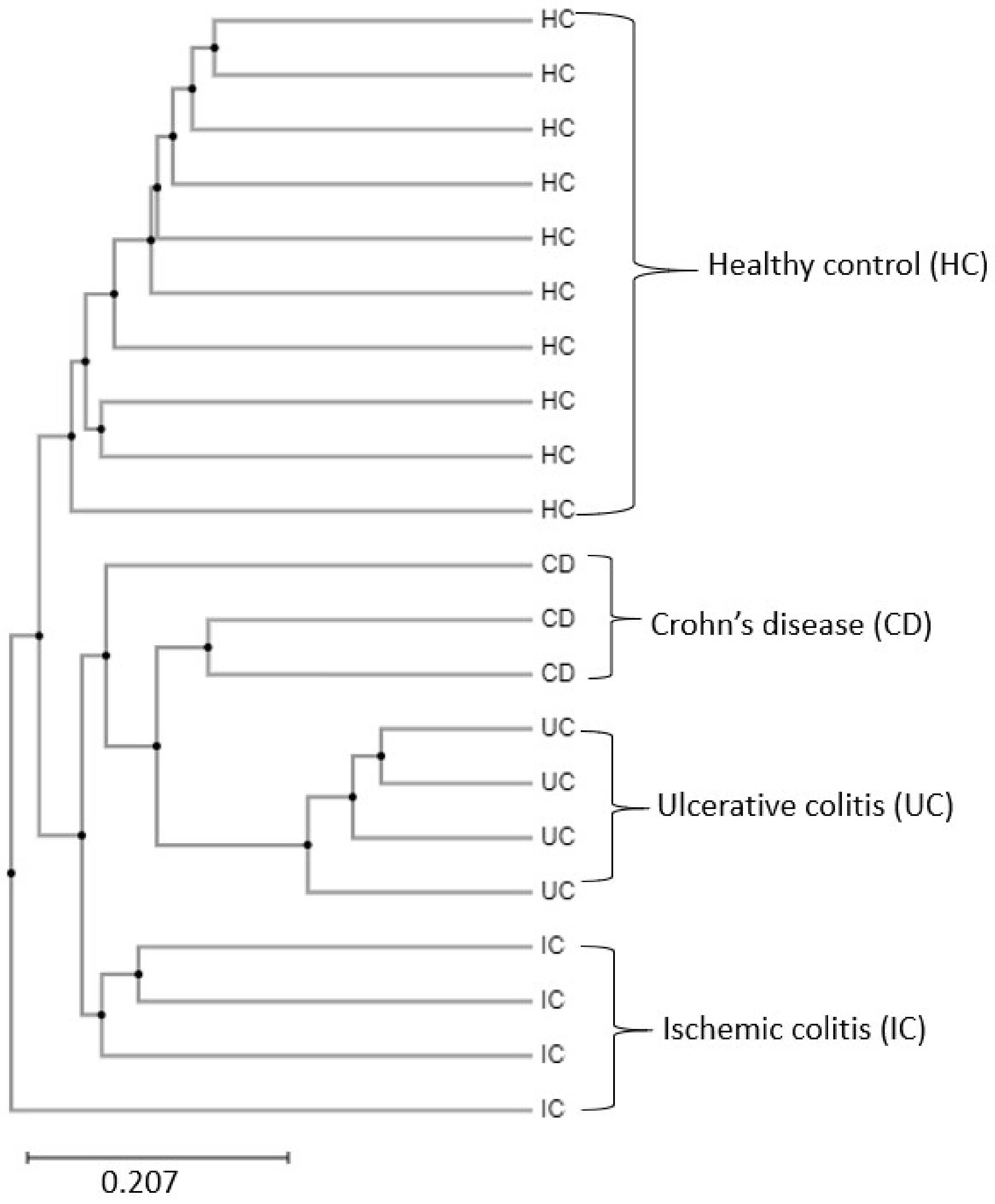

Supplementary Figure S19. Beta diversity of HC, CD, UC, IC samples. Phylotypes were clustered with UPGMA (unweighted pair group method with arithmetic mean) based on generalized UniFrac beta diversity distance including species rank and unclassified OTUs without normalization.

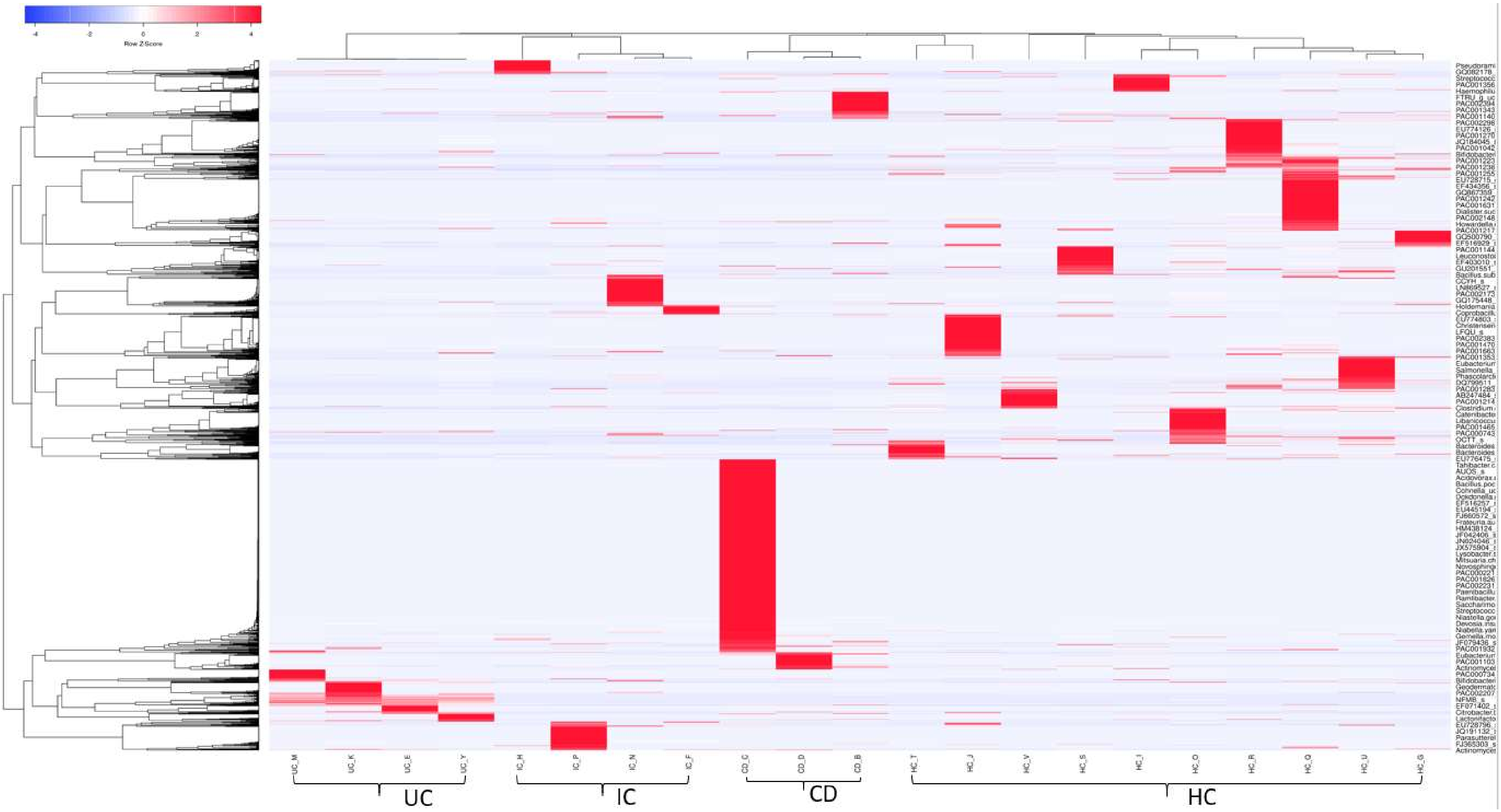

Supplementary Figure S20. Expression heatmap at the species level with the complete linkage of clustering and Pearson's distance measurement method.

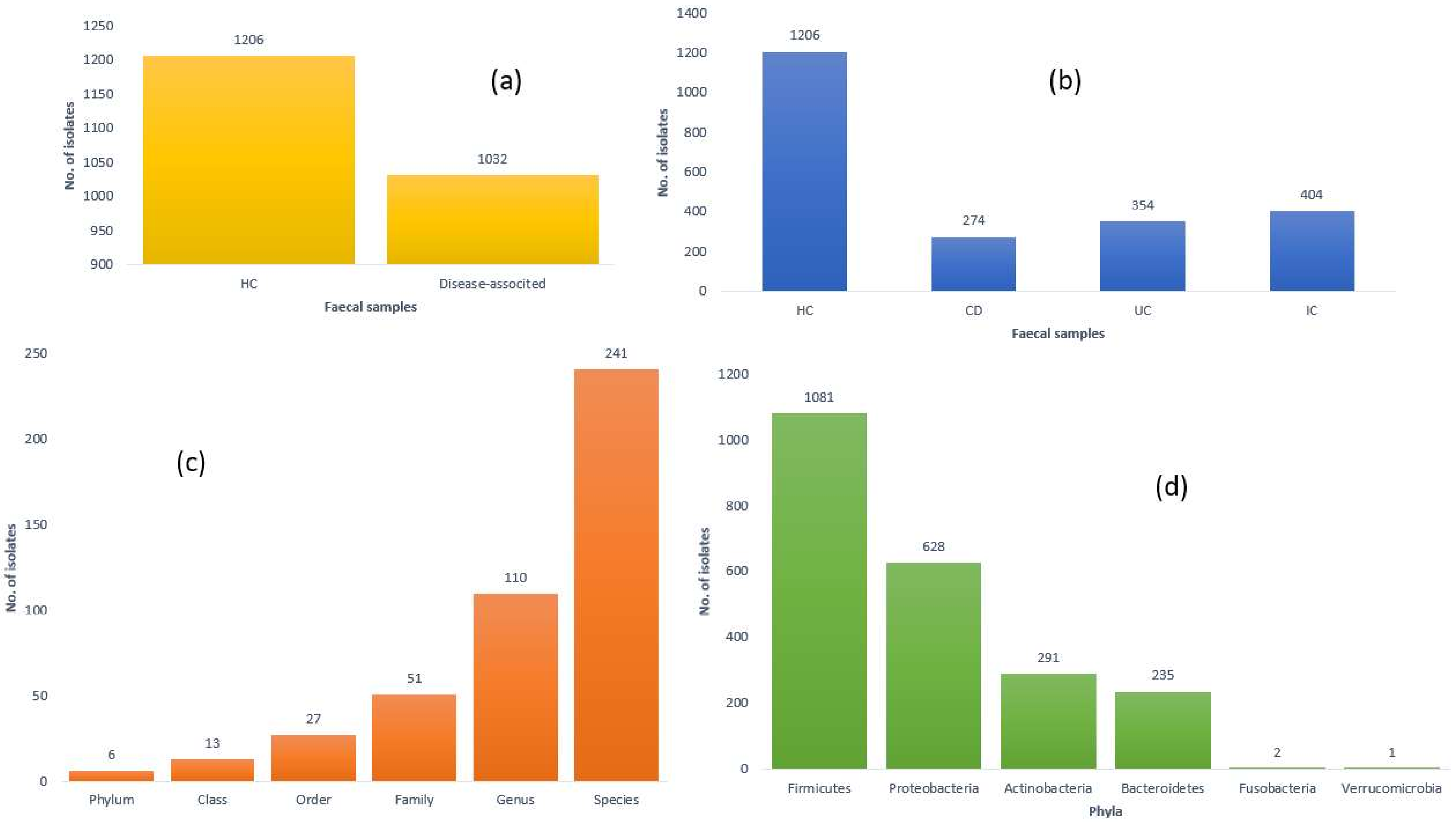

Supplementary Figure 21. Total number of bacteria isolated from each sample in disease-associated conditions (IBD and IC) and healthy control (a) and (b); total number of phyla, classes, orders, families, genera, and species (c); and species abundance at different phylum level (d).

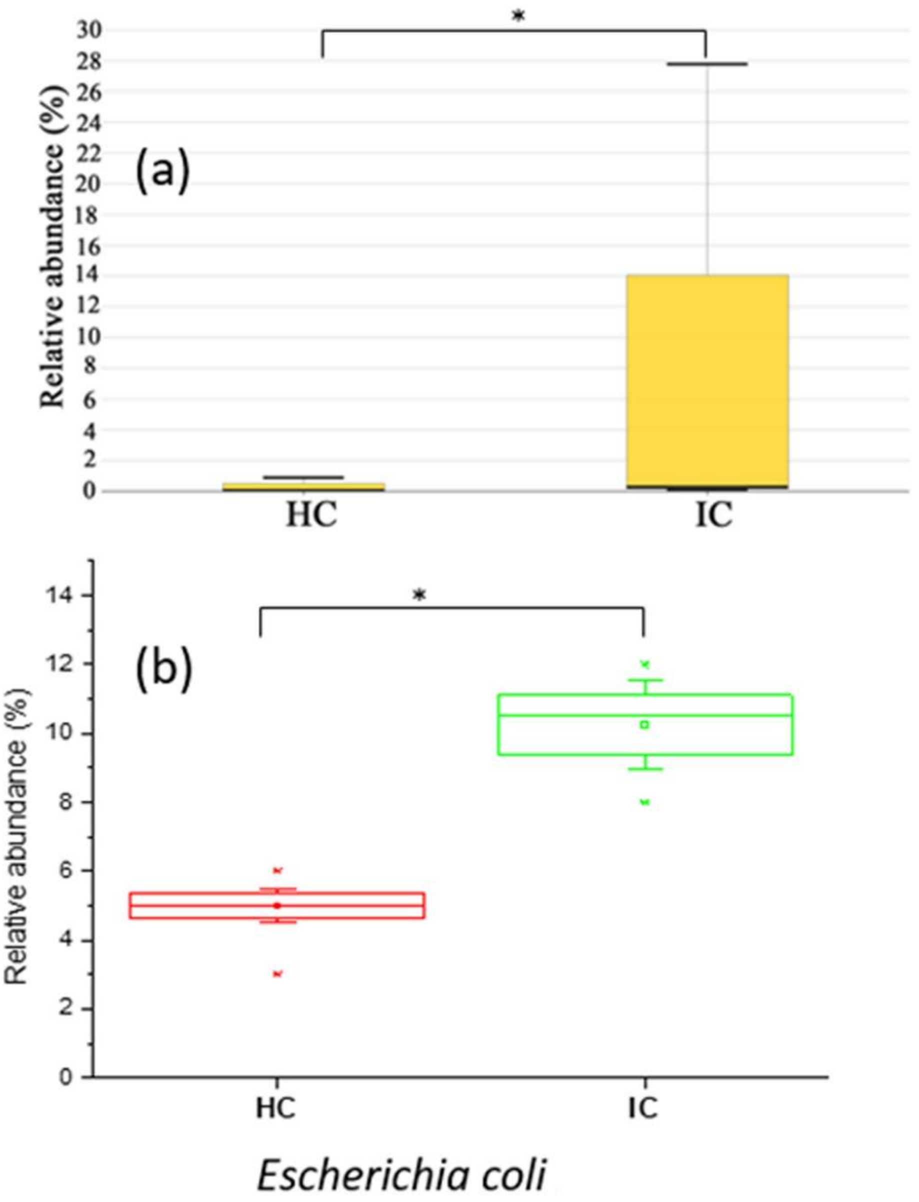

Supplementary Figure S22. Relative abundance of *Escherichia coli* in IC condition against HC. Data were from 16-based MTP (a) and culturomics (b). Statistical significance was measured based on Wilcoxon rank-sum test. *$P < 0.05$.
